# The Development of Positron Emission Tomography Tracers for In Vivo Targeting the Kinase Domain of the Epidermal Growth Factor Receptor

**DOI:** 10.3390/ph15040450

**Published:** 2022-04-05

**Authors:** Antonia Högnäsbacka, Alex J. Poot, Danielle J. Vugts, Guus A. M. S. van Dongen, Albert D. Windhorst

**Affiliations:** 1 Department of Radiology & Nuclear Medicine, Amsterdam UMC Location Vrije Universiteit Amsterdam, De Boelelaan 1117, 1081 HV Amsterdam, The Netherlands; a.j.poot@umcutrecht.nl (A.J.P.); d.vugts@amsterdamumc.nl (D.J.V.); gams.vandongen@amsterdamumc.nl (G.A.M.S.v.D.); 2 Cancer Center Amsterdam, Imaging and Biomarkers, 1081 HV Amsterdam, The Netherlands

**Keywords:** EGFR, PET tracer, evaluation

## Abstract

Multiple small molecule PET tracers have been developed for the imaging of the epidermal growth factor receptor (EGFR). These tracers target the tyrosine kinase (TK) domain of the receptor and have been used for both quantifying EGFR expression and to differentiate between EGFR mutational statuses. However, the approaches for in vivo evaluation of these tracers are diverse and have resulted in data that are hard to compare. In this review, we analyze the historical development of the in vivo evaluation approaches, starting from the first EGFR TK PET tracer [^11^C]PD153035 to tracers developed based on TK inhibitors used for the clinical treatment of mutated EGFR expressing non-small cell lung cancer like [^11^C]erlotinib and [^18^F]afatinib. The evaluation of each tracer has been compiled to allow for a comparison between studies and ultimately between tracers. The main challenges for each group of tracers are thereafter discussed. Finally, this review addresses the challenges that need to be overcome to be able to efficiently drive EGFR PET imaging forward.

## 1. Introduction

### 1.1. EGFR and the Erb Receptor Family

The epidermal growth factor receptor (EGFR, HER1/ErbB1) is a member of the ErbB receptor family that contains the structurally related ErbB2 (HER2/Neu), ErbB3 (HER3), and ErbB4 (HER4) [1]. ErbB1 and ErbB4 are autonomous receptors, while ErbB2 and ErbB3 do not bind soluble ligands but act as partners in heterodimeric complexes with the other receptors of the ErbB family [2]. Two models are suggested for the activation of ErbB1: the “ligand-induced dimerization model” and the “rotation model”. In the ligand-induced dimerization model, receptor dimerization is initiated after ligand binding. In the rotational model, however, the receptors exist in the dimeric form in a non-active state, but upon ligand binding, the conformation of the complex causes the receptor to activate [1,3].

There have been seven natural ErbB1 agonists reported—the epidermal growth factor (EGF), transforming growth factor α (TGFα), betacellulin (BTC), heparin-binding EGF-like growth factor (HB-EGF), amphiregulin (AREG), epigen (EPN), and epiregulin (EPR). Once the transmembrane receptor is activated, auto-phosphorylation of tyrosine residues within the cytoplasmic region of the receptor occurs. Depending on the binding ligand, different biological responses are subsequently induced such as cell differentiation, proliferation, and survival [1,4].

### 1.2. Role of EGFR in Cancer

In 1984, EGFR was extracted and purified from both the human epidermoid carcinoma cell line A431 and human placenta. It was discovered that an oncogenic avian erythroblastosis retrovirus encoded a protein sequence similar to EGFR [5]. This led to further interest in elucidating the connection between the EGF receptor and cancer development. Elevated levels of EGFR expression and its ligands have been recognized in several cancers. Nicholson et al. compared more than 200 studies in an effort to find a relation between EGFR expression and cancer prognosis. Elevated levels of EGFR relative to normal tissues were found for ten cancer types. Additionally, in head and neck, ovarian, cervical, bladder, and esophageal cancer, elevated EGFR levels correlated with poor patient prognosis. Meanwhile, in gastric, breast, endometrial, and colorectal cancer, the elevated level did not correlate as strongly to patient outlook. In non-small cell lung cancer (NSCLC), the EGFR expression level was rarely related to overall and relapse-free survival [6].

With the importance of EGFR expression in cancer being evident, several approaches to inhibit EGFR have been undertaken. Extracellular inhibition utilizes monoclonal antibody-based drugs that are able to compete with the EGFR ligands. Intracellular inhibition can be achieved by targeting the ATP-binding site of the tyrosine kinase domain of the receptor, giving the name tyrosine kinase inhibitors (TKIs, for further information on inhibition strategies, see [7]). However, despite a high expression level of EGFR, a subset of cancers did not respond to anti-EGFR treatment [8,9,10]. Receptor mutations offered a possible explanation for the difference in sensitivity to EGFR inhibition. The first mutation in the EGFR gene was found in human gliomas, where amplification and overexpression of EGFR were frequent. This deletion in the EGFR gene led to truncated EGFR proteins, the most prevalent being the EGFRvIII mutation [11,12]. 

These truncated EGFR proteins have been seldom observed in NSCLC, so the overexpression or amplification of EGFR in NSCLC cannot be explained by these extracellular mutations observed in human gliomas [13,14,15]. Somatic mutations, resulting in alterations in the tyrosine kinase domain of the EGFR receptor, were later discovered and linked to gefitinib treatment response. The first substitution mutations reported were L858R, G719S, and L861Q, along with multiple deletion mutations in the region of codons 746 to 759 (Del19) within the kinase domain. The L747–P753insS deletion as well as the L858R mutation were demonstrated to lead to increased auto-phosphorylation in Cos-7 cells compared to wild-type EGFR expressing cells. It was also shown that in the wild-type EGFR expressing cells, the activation of EGFR was downregulated after 15 min, which was consistent with the internalization of the receptor. In contrast, the activation was continued for up to 3 h for the mutant cell lines, even though the receptor expression level was equal in the mutated and wild-type cells [16,17]. These observations led to the development of tyrosine kinase inhibitors specific for the inhibition of mutated tyrosine kinases. The most commonly occurring activating mutation in NSCLC patients is the substitution mutation L858R in exon 21 and the deletion mutations in exon 19 (Del19). Patients with these mutations usually respond well to treatment with first-generation small-molecule TKIs such as gefitinib and erlotinib [16,17,18]. Nonetheless, patients initially benefitting from TKI treatments invariably develop treatment resistance to these drugs in the form of mutations such as HER2 mutation and amplification, MET amplification, PIK3CA mutation, BRAF mutation, NF1 loss, and potentially FGFR signaling [19,20]. Treatment resistance in the form of additional EGFR mutations has also been reported, the most common mutation being T790M, occurring in exon 20, which is an additional mutation in the EGFR kinase domain, and thought to be able to reactivate the protein [21,22]. To circumvent resistance or increase activity against the T790M mutation, second-generation and covalently binding TKIs such as afatinib, neratinib, and dacomitinib have been developed [23,24]. However, treatment resistance development has also been observed for second-generation EGFR inhibitors [25,26,27]. To inhibit T790M mutated EGFR, third-generation EGFR inhibitors such as osimertinib and rociletinib have been developed. Despite their potency against T790M mutated EGFR, the third generation of TKIs also give rise to a resistance mutation in the form of C797S [28]. Due to this recurring treatment resistance pattern, new kinase inhibitors are continuously being developed. Some of these (e.g., EAI045) work by allosteric inhibition of the auto-phosphorylation domain [27,29]. 

### 1.3. PET Imaging

Positron emission tomography (PET) is a nuclear imaging technology extensively used for diagnosis, therapy response prediction and monitoring, and drug development. The technology utilizes molecules labeled with positron-emitting radionuclides such as carbon-11 or fluorine-18, and are called radiopharmaceuticals or radiotracers. The distribution of a tracer inside the body is imaged with a PET camera, and the result is used to monitor a particular biological process or the presence of biological markers. For example, radiotracers such as [^18^F]FDG can illustrate the uptake and metabolism of glucose in tissues, while radiotracers such as [^68^Ga]Ga-PSMA-11 specifically target the prostate-specific membrane antigen, illustrating the level of antigen present in the tissue. [30]

Following the discovery of EGFR and the association of expression levels with clinical prognosis, a reliable way of quantifying the expression of EGFR in a tumor was sought. Immunohistochemistry (IHC) staining was thought to be unreliable for the quantification of EGFR for multiple reasons such as tumor heterogeneity, lack of sensitivity in the detection system, and challenges in attaining the sample [31]. Therefore, multiple small molecule PET tracers have been designed and developed to quantify EGFR expression, thereby offering a non-invasive alternative to immunohistochemistry and the necessity of obtaining biopsies. When somatic mutations were discovered, which explained the difference in response to TKI treatment in NSCLC patients, the ability of TKI PET tracers to selectively target mutated EGFR was more extensively evaluated.

This review describes the path toward small molecule PET tracers for the imaging of the EGFR kinase domain and discusses the successes and pitfalls in preclinical and clinical EGFR TKI PET research.

## 2. EGFR TKI PET Tracer Development

**Figure 1:**
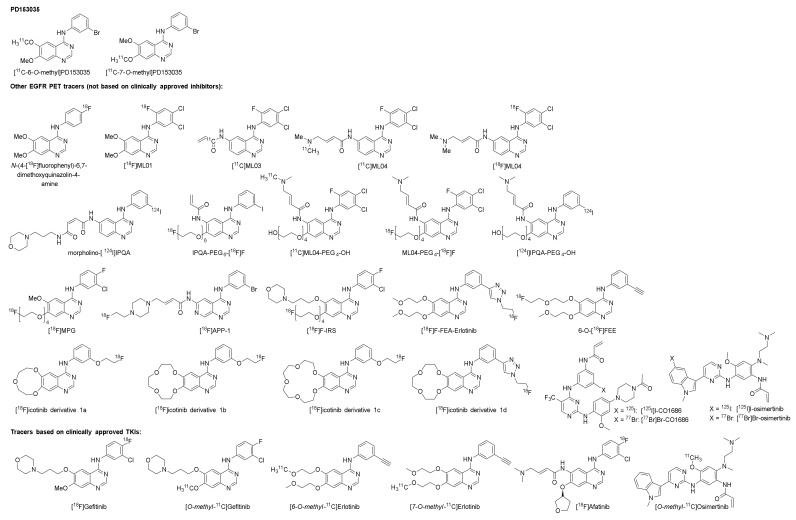
EGFR tyrosine kinase targeting PET tracers presented and discussed in the review.

The following section provides an overview of EGFR tyrosine kinase PET tracers that have been evaluated in vitro and/or in vivo. The tracers are described in three sections (Figure 1). The first section describes the first extensively studied EGFR TKI PET tracer, [*O-methyl*-^11^C]PD153035. The second section describes other compounds developed for the purpose of imaging EGFR tyrosine kinases. Finally, the third section describes EGFTK PET tracers based on clinically approved TKIs.

### 2.1. [^11^C]PD153035

PD153035 was discovered among a series of ATP competitive TKIs. The compound was shown to be selective toward EGFR tyrosine kinase when compared to other tyrosine kinases such as the platelet-derived growth factor receptor, the fibroblast growth factor receptor, the colony-stimulating factor-1 receptor, insulin receptor, and src tyrosine kinases. Only for the structurally related HER2 (ErbB2) receptor was a discernable inhibition observed, however, at a ~100,000-fold lower IC_50_ value than for EGFR (2.3 µM vs. 29 ± 5.1 pM). The potency and selectivity of PD153035 were also evaluated using viable Swiss 3T3 fibroblasts. PD153035 suppressed tyrosine phosphorylation upon EGF induction, while this was not observed when the fibroblasts were induced by PDGF or basic FGF [32].

**Figure 2:**
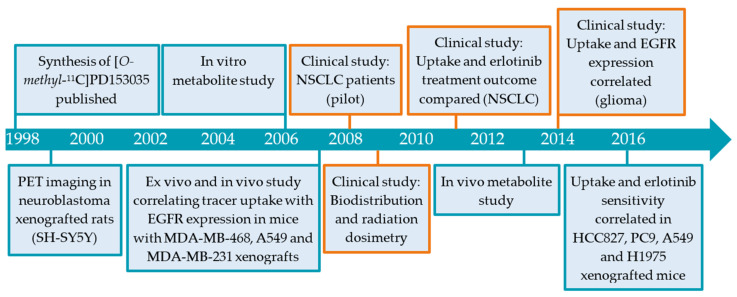
Studies involving [*O-methyl-*^11^C]PD153035 (blue outline: preclinical, orange outline: clinical).

To study the behavior of PD153035 in vivo, [*O*-*methyl-*^11^C]PD153035 was developed and was thereby the first TKI-PET tracer published for imaging of the EGFR kinase domain [33,34]. Figure 2 presents a chronological overview of the different preclinical and clinical studies that have been carried out from 1998 to 2016 using [*O*-*methyl-*^11^C]PD153035 and will be discussed in this review.

#### 2.1.1. Preclinical Development of [*O-methyl*-^11^C]PD153035

**Figure 3:**
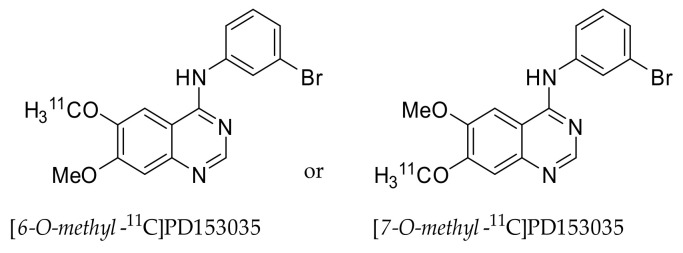
The different labeling positions for [*O-methyl-*^11^C]PD153035.

The synthesis of [*O-methyl*-^11^C]PD153035 was first published in 1998. The method used did not allow for determination if the labeling occurred at the 6- or 7-*O-methyl* position. Demethylated PD153035 was methylated using [^11^C]CH_3_I, resulting in [*O-methyl*-^11^C]PD15303 (Figure 3) [35]. The biodistribution of the resulting tracer in the blood, brain, gastrointestinal tract, and liver was evaluated by PET in a single female Sprague Dawley rat while taking arterial samples. The tracer was shown to be quickly cleared from the plasma [36]. The rapid clearance of radioactivity from plasma in rats was also observed by Samén et al. [37], and in female BALB/c nude mice by Wang et al. [38].

The first evaluation of the metabolism of [*O-methyl*-^11^C]PD153035 was carried out by incubating [6-*O-methyl*-^11^C]- and [7-*O-methyl*-^11^C]PD153035 with human and rat liver microsomes and comparing the metabolites. Some species differences were observed in the evaluation. The percentage of the main metabolite “M1”, proposed to be an oxidized analog, was larger when incubated with rat liver microsomes than with human liver microsomes. The occurrence of 7-O-demethylated PD153035 was also markedly more prevalent when using human liver microsomes compared to that of rats (25% versus 4%) [39]. The in vivo evaluation of the metabolism of [6-*O-methyl*-^11^C]PD153035 in male Sprague-Dawley rats indicated rapid tracer metabolism, with 5% intact tracer present in plasma 30 min post-injection. The most abundant radiometabolites in the plasma were polar metabolites, but contrary to the in vitro study, the 7-O-desmethyl metabolite could be detected in >20% of the total [37]. The involvement of CYP3A and CYP2D P450 enzymes in the metabolism of [6-*O-methyl*-^11^C]PD153035 was further investigated in in vivo studies where CYP3A and CYP2D enzymes were inhibited using ketoconazole (10 mg/kg) or quinidine sulfate dihydrate (20 mg/kg). Compared to untreated animals, rats treated with ketoconazole had a 3-fold higher level of intact tracer. In comparison, rats treated with quinidine had a 2-fold higher level of intact tracer, illustrating the involvement of CYP3A and CYP2D in the metabolism of the tracer [37].

Multiple preclinical studies have evaluated the tumor-targeting potential of [*O-methyl*-^11^C]PD153035 (Table 1) but in many of the studies, the location of the carbon-11 has not been defined. The first study evaluating the tumor-targeting potential of [*O-methyl-^11^C*]PD153035 was carried out in immunodeficient male WAF rnu/rnu rats xenografted with the neuroblastoma cell line SH-SY5Y observed to express high levels of EGFR. An initial increase in tumor uptake (up to 0.23–0.33% ID/mL at 8 min) was observed, followed by a decline in radioactivity concentration [36]. The correlation between [*O-methyl*-^11^C]PD153035 accumulation and EGFR expression levels (as assessed by immunohistochemistry) was also evaluated in the breast cancer cell lines MDA-MB-468 and MDA-MB-231 and the lung cancer cell line A549 xenografted in female BALB/c nude mice. Similar to the neuroblastoma study, the highest tumor uptake was observed at 10 min post-injection and strongly decreased thereafter [38,40]. Samén et al., reported a similar observation for [6-*O-methyl*-^11^C]PD153035 in A431 xenografted immunocompromised NIH-Foxn*1^rnu^* rats, the cell line from which EGFR was first isolated. The maximum uptake was observed at 2–3 min post-injection [37].

The ability to target EGFR mutations was also evaluated in four human NSCLC cell lines xenografted in female BALB/c nude mice. Tumor targeting was assessed by PET/CT and the uptake was compared to that of the contralateral muscle tissue and reported as the tumor-to-non-tumor ratio (T/NT). The highest T/NT ratio was achieved in the Del19 EGFR expressing xenografts HCC827 and PC9. The T/NT ratio peaked in HCC827 xenografts at 40 min post-injection, while the ratio in PC9 xenografts peaked at 25 min, albeit at a significantly lower level. The T/NT ratios in the wild-type EGFR expressing A549 and L858R/T790M expressing H1975 xenografts were lower than in the PC9 and HCC827 xenografted mice. The uptake in the xenografts correlated with the expression level of phosphorylated EGFR (pEGFR), as determined by western blot, but not with the EGFR expression level [42].

#### 2.1.2. Clinical Evaluation [*O-methyl*-^11^C]PD153035

**Figure 4:**
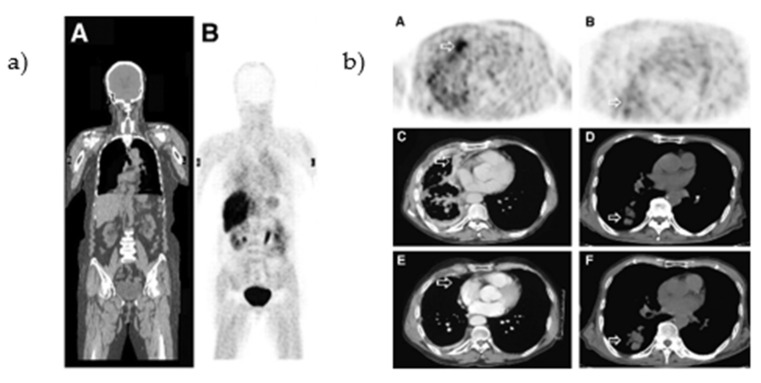
(**a**) [*O-methyl*-^11^C]PD153035 whole-body distribution 30–40 min after injection in a healthy volunteer. Adapted from [44], with permission from SNMMI. (**b**) Baseline [*O-methyl*-^11^C]PD153035 PET in (**A**) an adenocarcinoma patient and (**B**) a squamous cell carcinoma patient, with corresponding CT before treatment (**C**,**D**) and 6 weeks after treatment (**E**,**F**). Adapted from [46], with permission from SNMMI.

The biodistribution of [*O-methyl*-^11^C]PD153035 was evaluated in healthy human volunteers. At 60 min post-injection, the highest uptake was observed in organs connected to the renal and hepatobiliary system (bladder, gallbladder, kidneys, small intestine, and liver, Figure 4a) [43,44]. In a pilot study, the uptake of [*O-methyl*-^11^C]PD153035 was correlated to the EGFR expression levels (determined by immunohistochemistry and western blot) in gliomas. The majority of the glioblastomas could be visualized with PET with a SUV_ma__x_-to-white-matter ratio of 1.77 ± 0.41 [45]. In addition, Meng et al., correlated the tumor uptake to the efficacy of erlotinib therapy (mutational status of the tumors was not established). A baseline PET/CT scan was performed in 21 patients with adenocarcinoma or squamous cell carcinoma before the initiation of erlotinib treatment (Figure 4b). One to two weeks after the initiation of treatment, a second PET/CT scan was performed. The SUV_max_ at baseline and the second scan correlated with the overall survival (OS) and progression-free survival (PFS). In contrast, a third scan performed six weeks after treatment initiation did not correlate to either prognostic indicator [46].

#### 2.1.3. Limitations in [*O-methyl*-^11^C]PD153035 Evaluation Interpretation

A major limitation in the interpretations of the [*O*-*methyl*-^11^C]PD153035 studies is due to the uncertainty of the position of the carbon-11 radionuclide. In the study conducted by Fredriksson et al., in 1999, [*O-methyl*-^11^C]PD153035 was prepared by reacting [^11^C]CH_3_I with a precursor prepared via mono-demethylating PD153035 [36]. The position of the label was not known at this stage, as was openly reported in the article. No information about the precursor or the position of the label was given in the articles of Wang et al. [38,40], Liu et al. [44], Meng et al. [46], Sun et al. [45], and Dai et al. [42]. Most of these articles refer, if not directly, to the article of Fredriksson et al. The same research group eluded in a later publication that through their method, [6-*O-methyl*-^11^C]PD153035 was synthesized [39]. Therefore, it can only be assumed that the clinical studies were carried out using [*6-O-methyl*-^11^C]PD153035. The in vitro studies of Samén et al., showed that [*6-O-methyl*-^11^C]PD153035 is rapidly metabolized to 7-*O*-demethylated [*6-O-methyl*-^11^C]PD153035 by incubation with human liver microsomes [39]. It was also shown that in Sprague-Dawley rats, a major metabolite was the 7-*O*-demethylated [*6-O-methyl*-^11^C]PD153035 [37], which has been reported to have a similar affinity for EGFR as the parent compound (K_i_ value between 25 pM and 168 pM, compared to 6 pM for PD153035) [47]. Considering these K_i_ values, it is possible that the 7-*O*-demethylated [*6-O-methyl*-^11^C]PD153035 metabolite contributes to the overall uptake in tumors, but off-target affinity is equally likely. Furthermore, the percentage of intact [*6-O-methyl*-^11^C]PD153035 in Sprague-Dawley rats was shown to rapidly decrease to approximately 10% within 10 min [37]. However, no information on the metabolism of the tracer in humans has been reported, which makes it difficult to interpret the results and assess the ability of [*O-methyl*-^11^C]PD153035 to target tumors. 

### 2.2. Other EGFR TKI PET Tracers (Not Based on Clinically Approved Inhibitors)

**Figure 5:**
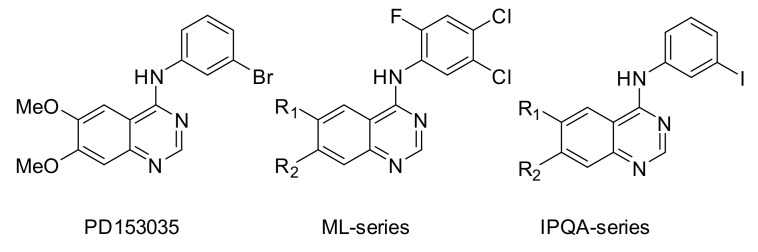
The core structure of PD153035, ML-, and IPQA-compounds.

**Figure 6:**
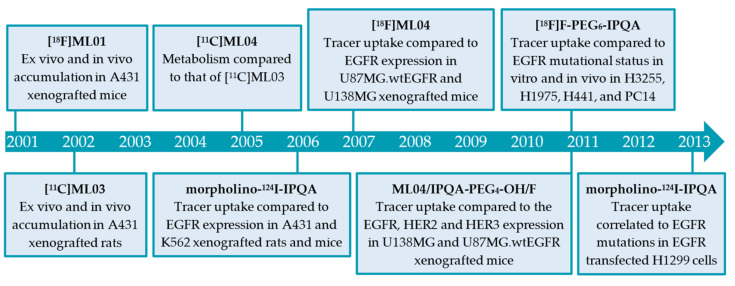
Timeline over the development of the ML- and IPQA-tracers.

Carbon-11 has a half-life of 20 min, which limits the feasible imaging time. Prolonging imaging time may result in improved tumor-to-background ratios, as the unbound tracer and metabolites have more time to clear the biological system. In order to increase imaging time, other radionuclides such as fluorine-18 (half-life of 110 min) and iodine-124 (half-life of 100 h) were used in the development of the ML-series (Section 2.2.1) and the IPQA series (Section 2.2.2), respectively (Figure 5). The section below will discuss the development and evaluation of these series (Figure 6), along with some other EGFR TKI PET tracers derived from clinically approved inhibitors (Section 2.2.3).

#### 2.2.1. The ML-Series

**Figure 7:**
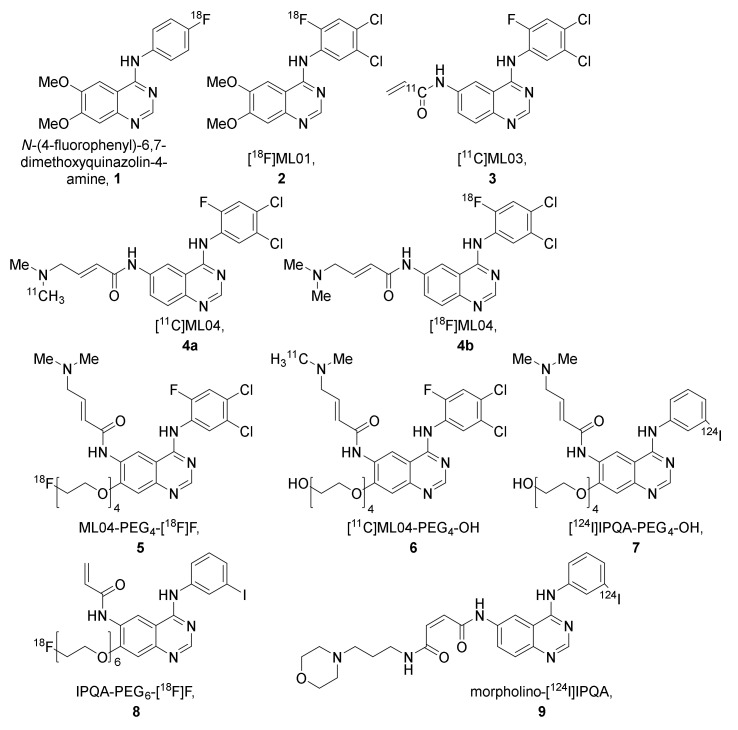
ML- and IPQA-tracers(**1**–**6** and **7**–**9**, respectively) discussed in the review.

The ML-series shares the same anilinoquinazoline scaffold as PD153035 and was developed to allow labeling with fluorine-18. In the first publication describing the series, several derivatives were synthesized and evaluated in vitro. Two of the most promising compounds, N-(4-[^18^F]fluorophenyl)-6,7-dimethoxyquinazolin-4-amine (**1**, Figure 7) and [^18^F]ML01 (**2**, Figure 7), were further evaluated in A431 xenografted male BALB cBy nu/nu mice [48].

Rapid blood clearance was observed for both **1** and **2**. Following blood collection, the blood was extracted and the intact tracer fraction was determined by radio-TLC. For **1,** 16% of the radioactivity in the blood could be extracted at 20 min post-injection, of which 75% was determined to be intact tracer. For **2,** on the other hand, 36% of the radioactivity in the blood could be extracted at 10 min post-injection, of which 56% was determined to be intact tracer (Table 2) [48]. 

The uptake in A431 tumors was evaluated at 30 and 60 min for **1**. The tumor uptake was observed to be higher at the later time point. Evaluation of **2** in vivo (PET for 60 min) and ex vivo at 5-, 10-, and 30-min post-injection revealed that in contrast to **1**, the tumor uptake peaked at the earlier time points (Table 2). Partial blocking of uptake of **2** could be achieved upon co-injection with the non-labeled compound. Compound **2** was extensively evaluated in vitro in A431 cells and, although indicated to have a high affinity for EGFR TK (Table 3), the binding potential determined did not indicate a successful tracer for PET [48]. 

To improve tumor retention, [^11^C]ML03 (**3**) was developed as a derivative of **2** with an acryloyl amide incorporated for irreversible binding (Figure 7). The ability of irreversible binding was demonstrated in A431 cells, where the compound successfully suppressed the kinase activity up to 8 h after the removal of the inhibitor (Table 3). The compound was labeled with carbon-11, and evaluated in male WAG rnu/rnu rats. Similar to **1** and **2**, fast clearance of **3** was observed. At 60 min post-injection, 17% of the radioactivity in the blood could be extracted, of which 75% was determined to be intact tracer (Table 2). The ex vivo evaluation of tumor-targeting revealed only a low tumor uptake of the tracer that could not be visualized by PET imaging, which decreased over time (Table 2) [49]. 

It was hypothesized that **3** was chemically too reactive, which led to rapid in vivo metabolism of the compound. Further modifications were made to the scaffold to improve the bioavailability and retention, which led to the discovery of ML04 (**4**, Figure 7). The compound was labeled with carbon-11, resulting in [^11^C]ML04, **4a**. The metabolism of tracer **4a** was compared to that of **3** in male nude Hsd:RH-rnu/rnu rats. Contrary to **3,** no metabolites were detected for **4a** in the extracted blood fraction at 60 min post-injection (Table 2) [50]. 

A ^18^F-labeled version of compound **4** was developed, **4b**, to evaluate the ability of the compound to differentiate between EGFR expression levels. NUDE-Hsd athymic nude nu mice were xenografted with U87MG.wtEGFR, a cell line expressing a high level of EGFR, or U138MG, a cell line lacking EGFR expression. The tumor uptake was evaluated at 60-, 120-, 180-, and 240-min post-injection in U87MG.wtEGFR xenografts. The highest uptake was observed at the 60-min time point, but the highest tumor-to-tissue ratio was achieved at 180 min post-injection (Table 4). In contrast, although lower than in U87MG.wtEGFR, the measured activity remained stable in U138MG xenografts over the three-hour time period (Table 4). A slight decrease in uptake could be observed in U87MG.wtEGFR xenografts following pretreatment with the cold compound (5–8 mg/kg). Extensive in vitro evaluation was conducted to evaluate the tracer’s potency, selectivity, and irreversible binding (Table 3). The compound showed inhibition of EGFR expressing cell lines at low concentrations and selectively inhibited the EGFR compared to other kinase receptors. However, the affinity for the ErbB family receptor HER2 was comparable to that of EGFR. The ability of **4b** to quantify EGFR in intact A431 cells was examined in vitro, and though **4b** showed promise, an affinity to other macromolecules is likely [52].

To further improve the solubility and reduce the lipophilicity of compound **4**, polyethylene glycol (PEG) chains of various lengths were introduced. For example, by adding a fluoro-polyethyleneglycol (PEG_4_-F) chain to compound **4**, resulting in ML04-PEG_4_-[^18^F]F (**5**, Figure 7), the lipophilicity was decreased from LogP = 3.9 to 3.7 and the solubility was significantly increased from 0.14 µg/mL to 3.5 µg/mL, while retaining the irreversible binding capacity (Table 3) [51,67]. In vivo evaluation of **5**, along with hydroxyl polyethylene glycol ML04 derivative, [^11^C]]ML04-PEG_4_-OH, (**6**) in U87MG.wtEGFR and U138MG xenografted athymic Crl:CD-1-Foxn1nu/nu mice, demonstrated that on average, the tumor uptake did not indicate any retention over time and no significant difference in uptake of the tracers was observed between the U138MG and U87MG.wtEGFR xenografts. The EGFR, HER2, or HER3 expression could not explain the similar uptake in the two xenograft lines. The ratio of activated EGFR to total EGFR was observed to be similar for the two xenograft lines, so it was suggested that future evaluation of these compounds should be carried out in EGFR mutation activated cells [53].

#### 2.2.2. The IPQA-Series

One of the derivatives developed in the same series as tracers **5** and **6** was [^124^I]IPQA-PEG_4_-OH (**7**) [51]. This tracer was also evaluated in U87MG.wtEGFR and U138MG xenografted athymic Crl:CD-1-Foxn1nu/nu mice. However, the tumor uptake was less than the background concentration in both tumors at the majority of the time points [53].

Adding a fluorinated PEG chain to the IPQA core resulted in IPQA-PEG_6_-[^18^F]F, (**8**, Figure 7). The compound was shown to preferentially inhibit L858R mutant EGFR kinases (IC_50_ = 92 nM) when compared to resistance mutated L858R/T790M EGFR kinases (IC_50_ = 4.4 mM) and wild-type EGFR kinases (IC_50_ = 1.1 mM) [54]. The ability of **8** to differentiate between EGFR mutational status was evaluated in vitro and in vivo. The metabolic stability of the tracer was evaluated in nu/nu mice bearing subcutaneous tumors: 16.2 ± 8.6% of intact tracer was observed in plasma at 30 min post-injection [54]. In both the in vitro and in vivo evaluation, the highest accumulation was observed in the L858R EGFR mutation expressing cell/xenograft line H3255 compared to that of H1975 (L858R/T790M), H441 (wild-type EGFR), and PC14 (low wild-type EGFR expression, Table 4). Both in vitro and in vivo, the tracer uptake in H3255 cells/tumors could be reduced by blocking with gefitinib [54,68].

Further modification was also performed on the functional group at the 6-position to lower the lipophilicity and reduce the hepatobiliary clearance, thereby improving the circulation halftime. One such compound was morpholino-IPQA (**9**, Figure 7). The compound was shown to inhibit the growth of U87MG (IC_50_ 0.38 μM), U87MG∆EGFR (IC_50_ 0.12 μM), and A431(IC_50_ 0.096 μM) cells and inhibit the EGFR phosphorylation with an IC_50_ of 0.6 nM. The compound was labeled with iodine-124 (**9**). The ability of **9** to target activated EGFR kinases was evaluated in vivo by PET in both rnu/rnu rats and in nu/nu mice xenografted with A431 (EGFR positive) or K562 (negative control). A time-dependent accumulation of radioactivity in A431 tumors was observed in both rats and mice, while this was not observed in the negative control K562 xenograft (Table 4) [62]. Morpholino-[^131^I]IPQA, labeled with iodine-131, was further evaluated in NOD/SCID mice bearing H1299 tumors transfected with wild-type EGFR, the L858R mutation, the Del19 (E746-A750del) mutation, and transfection vector to show that the tracer was suitable as an imaging agent to distinguish EGFR tyrosine kinase activating mutations. However, similar uptake, which decreased over time, was observed for all the xenografts (Table 4). Morpholino-[^124^I]IPQA, labeled with iodine-124, was evaluated in a PET study, where the authors reported the highest tumor accumulation to be observed 24 h after injection in L858R transfected tumor xenografts (Table 4) [63].

#### 2.2.3. Derivatives of PD153035, Gefitinib, Erlotinib, Icotinib, and Rociletinib

**Figure 8:**
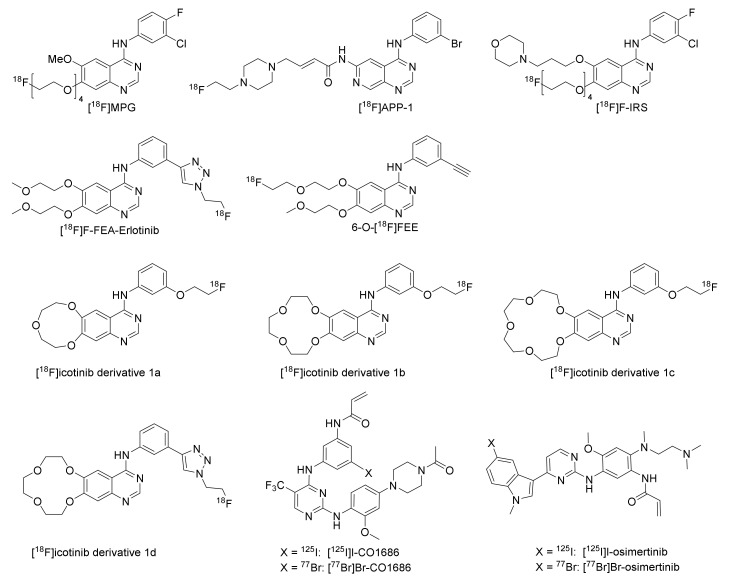
Additional EGFR TKI PET tracers discussed in Section 2.2.3.

Several derivatives of PD153035, gefinitib, erlotinib, icotinib, and rociletinib have been radiolabeled and explored as EGFR TKI PET tracers (Figure 8). A PEGylated analog of PD153035 was radiofluorinated, resulting in [^18^F]MPG, which showed preferential inhibition of the Del19 EGFR mutated HCC827 (IC_50_ 4.4 ± 1.1 nM) when compared to the EGFR resistance mutated H1975 (IC_50_ 21.4 ± 1.7 μM), EGFR-negative H520 (IC_50_ 10.1 ± 3.6 μM), and EGFR wild-type expressing H358 (IC_50_ 12.8 ± 7.5 μM). [^18^F]MPG was evaluated in vivo and ex vivo in female athymic nude mice xenografted with HCC827, H1975, H520, and H358 cells. The tracer was observed to be stable in plasma at one-hour post-injection (96% intact tracer). The highest uptake was observed in HCC827 xenografts both in vivo and ex vivo (Table 4). The T/M ratio for HCC827 was more than triple compared to the resistance mutated H1975, wild-type EGFR expressing H358, and EGFR-negative H520. The specificity was further evaluated by a blocking study in HCC827 xenografted mice. The uptake could be reduced twofold by administering 100 mg/kg gefitinib one hour before PET imaging. The diagnostic potential of the tracer was evaluated in 75 NSCLC patients. The highest organ radiation dose values were observed in the gallbladder, thyroid, bladder, and pancreas. A significant difference in SUV_max_ was observed between patients with EGFR activating mutation and those expressing wild-type EGFR. No significant decrease in [^18^F]MPG tumor uptake was observed in patients with EGFR activating mutations after TKI treatment. The SUV_max_ was also correlated to gefitinib treatment response, and it was showed that a greater response to gefitinib treatment was achieved when the [^18^F]MPG PET/CT SUV_max_ was ≥2.23 [56].

In order to facilitate differentiation between primary and secondary EGFR TK mutations, [^18^F]APP-1 was developed. It showed preferential inhibition of primary L858R EGFR kinases compared to secondary L858R/T790M EGFR kinases (IC_50_ 15.6 ± 0.8 nM vs. 326 ± 64 nM). The ability of the tracer to differentiate between primary and secondary EGFR TK mutations was evaluated with dynamic PET imaging by comparing the uptake in H3255 (L858R EGFR mutation) xenografts with the uptake in resistance mutated H1975 (L858R/T790M EGFR mutation) xenografts in BALB/c nude mice. A higher tumor-to-muscle ratio was observed in H3255 compared to H1975 xenografts (Table 4) and co-injection of osimertinib (17 µg) reduced the uptake by 50% in H3255 while no decrease of uptake in H1975 tumors was observed (Table 4) [57]. 

A derivative of gefitinib, [^18^F]F-IRS, containing a PEG_4_ chain at the 7-position, was developed to increase the water-solubility of the compound (Figure 8). The compound was evaluated in female BALB/c nude mice bearing HCC827 (Del19 mutated EGFR, IC_50_ 5.6 ± 0.2 nM), H1975 (L858R/T790M mutated EGFR, IC_50_ 2.27 ± 1.17 μM), H520 (EGFR-negative, IC_50_ 18.36 ± 2.21 μM), or H358 (wild-type EGFR, IC_50_ 7.05 ± 2.30 μM) xenografts. PET/CT and ex vivo biodistribution studies showed the highest uptake to occur in HCC827 xenografts at 120 min post-injection (Table 4), which was in agreement with in vitro evaluation. The uptake in HCC827 xenografts could be reduced four-fold by pretreatment with gefitinib (100 mg/kg). In addition, the tracer was evaluated in three NSCLC patients with confirmed Del19 mutations. A SUV_max_ value of 2.44 ± 0.49 was observed in these tumors [58].

A fluorinated derivative of erlotinib containing a 1,2,3-triazole scaffold, [^18^F]F-FEA-erlotinib (Figure 8), was investigated to assess its capability to differentiate between EGFR mutational status in nude mice bearing HCC827 (Del19 mutated EGFR), HepG2 (low EGFR expression), or A431 (high EGFR expression) xenografts by PET. Similar to the in vitro evaluation, the highest tumor uptake was observed in the HCC827 xenografts compared to HepG2 and A431 xenografts (Table 4). The uptake in HCC827 xenografts could be reduced by pretreatment with 100 mg/kg erlotinib [64].

Another erlotinib derivative, 6-O-[^18^F]fluoroethylerlotinib (6-O-[^18^F]FEE, Figure 8), was also developed for the differentiation between EGFR mutational statuses. The metabolic stability of the tracer was evaluated in male BALB/c OlaHsd mice. Analysis of radioactivity present in plasma revealed over 96% intact tracer at 2-, 15-, and 30-min post-injection as determined by radio-TLC, with an extraction efficiency of the total radioactivity from the blood of 49 ± 14%. The ability of the tracer to distinguish between EGFR mutated and non-mutated tumors was determined in male Hsd: Athymic Nude-Fox1nu mice xenografted with HCC827 (Del19 mutated EGFR, IC_50_: 9 ± 9 nM), H1975 (L858R/T790M mutated, IC_50_: 3.6 ± 1.5 µM), and QG56 (wild-type EGFR, IC_50_: 14.4 ± 4.6 µM). The tumor uptake was two- to three-fold higher in HCC827 tumors compared to H1975 and QG56 tumors (Table 4). The radioactivity was retained in HCC827 and H1975 tumors, whereas a slow decline in radioactivity concentration was measured in QG56 tumors. A tendency of reduction in uptake in HCC827 xenografts upon pre-administration of erlotinib (6.4 ± 0.4 mg/kg) was observed [59]. 

Another first-generation TKI inhibitor is icotinib, which was approved for first-line treatment of somatic EGFR mutated NSCLC in China in 2014 [69]. Three icotinib derivatives labeled with fluorine-18 were developed, of which two had alterations to the crown ether ring, either bigger to promote faster clearance from blood ([^18^F]icotinib derivative 1a) or smaller to reduce the steric hindrance ([^18^F]icotinib derivative 1c, Figure 8). The tracers were evaluated in female Kunming mice bearing S180 xenografts. [^18^F]icotinib derivative 1b (Figure 8) showed the highest tumor uptake and tumor-to-blood and -muscle ratios (Table 4) at 30 min post-injection [65].

Another derivative of icotinib, [^18^F]icotinib derivative 1d (Figure 8), has a 1,2,3-triazole ring incorporated into the structure. The tracer was evaluated in male athymic nu/nu nude mice implanted with A549 xenografts. Tumor uptake of 0.90 ± 0.24%ID/g was observed at 90 min post-injection (Table 4). This uptake could be reduced three-fold upon icotinib pretreatment (0.1 mg) [66].

An iodinated derivative of the third generation TKI rociletinib, [^125^I]I-CO1686 (Figure 8), was developed for the monitoring of EGFR L858R/T790M mutations and to aid in patient selection before therapy with EGFR-TKIs. The cytotoxicity of I-CO1686 was established to be comparable to rociletinib in H1975 (IC_50_ 0.20 ± 0.05 μM) and H3255 (IC_50_ 0.50 ± 0.21 μM) cells. The stability of [^125^I]I-CO1686 was evaluated in phosphate-buffered saline and plasma and only slightly decreased during the 24-h incubation at 37 °C (~80% intact tracer). The ability of the tracer to differentiate between L858R (H3255) and resistance mutated L858R/T790M (H1975) xenografts was evaluated in tumor-bearing female BALB/c nu/nu mice. Similar tumor uptake was observed in H1975 and H3255 at one-hour post-injection, not matching the selectivity observed in vitro (Table 4). Furthermore, the uptake in the tumors was not significantly reduced by rociletinib pretreatment (30 mg/kg, i.p. injection one hour before tracer injection) [60].

As bromine has similar chemical properties to iodine but is smaller in size, which could be advantageous for interaction with the binding site, [^77^Br]Br-CO1686 (Figure 8) was developed. The cytotoxicity of Br-CO1686 was found to be comparable to that of I-CO1686 (IC_50_ 0.18 ± 0.06 μM in H1975 and 0.20 ± 0.01 μM in H3255 cells). The stability of [^77^Br]Br-CO1686 in phosphate-buffered saline and murine plasma was likewise comparable (>80% intact tracer after 24-h incubation at 37 °C). The ability of the tracer to differentiate between resistance mutation L858R/T790M expressing H1975 and wild-type EGFR expressing H441 xenografts was evaluated in tumor-bearing female BALB/c nu/nu mice. Tumor uptake was higher for [^77^Br]Br-CO1686 than [^125^I]I-CO1686, but it was assumed to be due to free radiobromide generated by debromination (Table 4) [55].

Halogenated derivatives of the third-generation TKI osimertinib, [^125^I]I-osimertinib, and [^77^Br]Br-osimertinib (Figure 8) were evaluated in vitro, and it was shown that the inhibition potency of the halogenated osimertinib derivatives was comparable to osimertinib in the EGFR L858R mutation expressing H3255 and the EGFR L858R/T790M resistance mutated H1975. The stability of the halogenated osimertinib derivatives was evaluated in phosphate-buffered saline and murine plasma. Over 80% of the tracers remained intact after a 24-h incubation at 37 °C in both conditions. The ability of the tracers to differentiate between L858R (H3255) and resistance mutated L858R/T790M (H1975) xenografts were evaluated in tumor-bearing male BALB/c nu/nu mice. The tumor uptake in H3255 xenografts was higher for both tracers than in H1975 xenografts at 4 h post-injection (Table 4). However, while the uptake of [^125^I]I-osimertinib in the H1975 xenografts could be significantly reduced by osimertinib pretreatment, it did not reduce uptake in H3255 xenografts (100 mg/kg, i.v. injection one hour before tracer injection) [61].

### 2.3. Isotopologue Labelled Tracers of Clinically Approved TKIs

**Figure 9:**
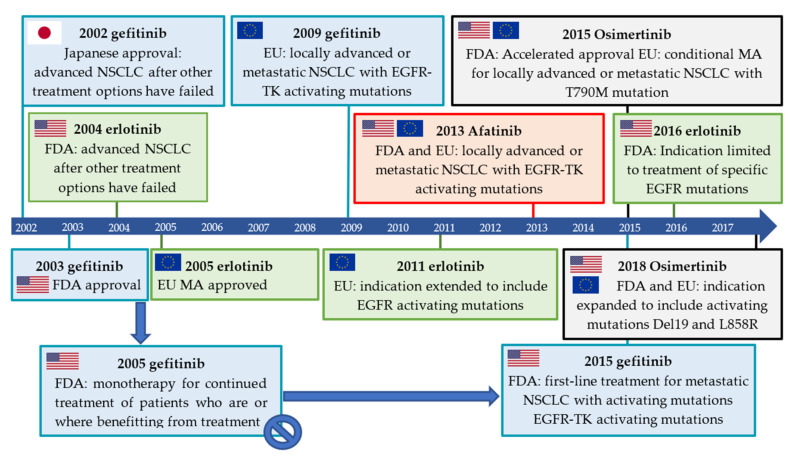
A timeline of the clinical approval and major decisions regarding EGFR targeting TKIs (gefitinib in blue, erlotinib in green, afatinib in red, and osimertinib in gray).

**Figure 10:**
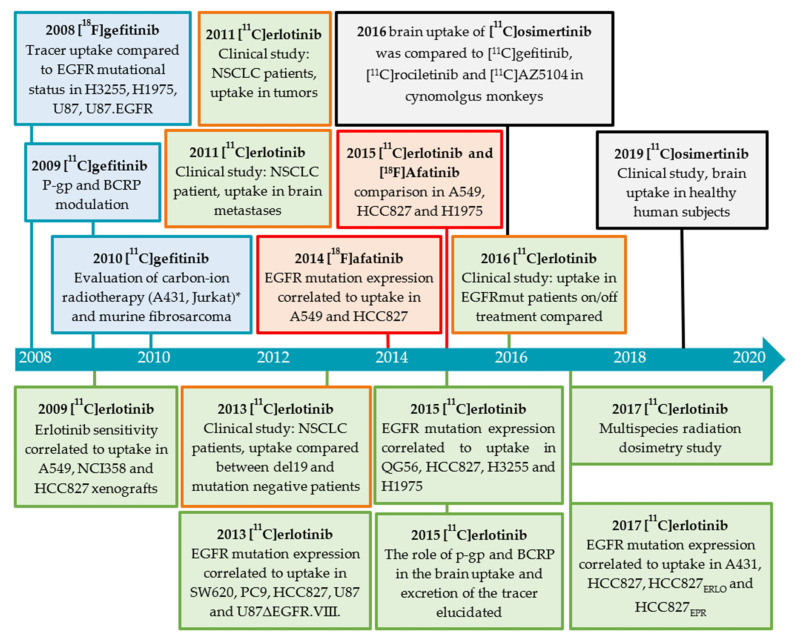
Studies involving gefitinib (blue), erlotinib (green: preclinical, orange: clinical), afatinib (red), and osimertinib (gray).

Gefitinib was the first EGFR inhibitor approved for the treatment of advanced NSCLC (Figure 9). However, erlotinib replaced gefitinib on the U.S. market when gefitinib failed to show an overall survival benefit in advanced NSCLC patients compared to the placebo in a confirmatory phase III trial [70,71,72,73]. While the discovery of EGFR activating mutations and their correlation to gefitinib treatment response led to the compound being approved for the treatment of NSCLC with activating TK EGFR mutations in the EU in 2009, it was only in 2015 that gefitinib returned to the U.S. market [16,17,23,74]. The indication for erlotinib was modified in 2016 by the FDA to limit the use of erlotinib from all NSCLC patients to patients with specific EGFR mutations [75]. The development and approval of these inhibitors are also reflected in the development and evaluation of the corresponding PET tracers (Figure 10).

The irreversibly binding, second-generation inhibitor afatinib received initial U.S. FDA approval and marketing authorization in the EU in 2013 as first-line treatment for patients with metastatic NSCLC with EGFR mutations (Del19 and L858R) [76,77]. Third-generation inhibitors still included the acryloyl amide, however, the 4-anilinoquinazoline core was now abandoned. In 2015, osimertinib received accelerated/conditional approval by the FDA and EMA for the treatment of patients with locally advanced or metastatic NSCLC expressing the resistance mutation T790M [78,79]. In 2018, this indication was expanded to include activating mutations Del19 and L858R (Figure 9) [80,81].

#### 2.3.1. Gefitinib

**Figure 11:**
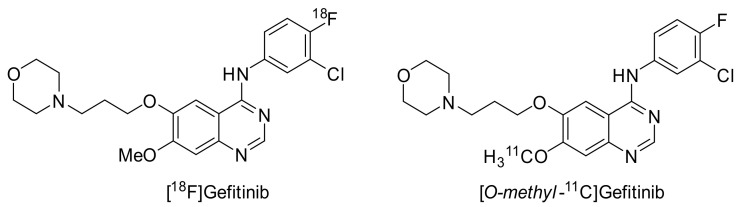
[^18^F]- and [*O-methyl-* ^11^C]gefitinib.

Gefitinib was selected from a library of 4-anilinoquinazolines, being metabolically stable and having a high affinity for EGFR. Gefitinib inhibited growth in a broad range of human solid tumor xenografts and demonstrated a long half-life in humans. The reversible inhibitor showed affinity for various tumor cells such as head and neck, breast, colorectal, ovarian, prostate, kidney, and glioma [82,83]. In order to understand the pharmacokinetics of gefitinib and to develop a tool to non-invasively determine the EGFR status of cancer cells in vivo, [^18^F]- and [*O-methyl*-^11^C]gefitinib were developed (Figure 11).

##### Preclinical Investigations

The stability of [^18^F]gefitinib was evaluated in non-tumor-bearing scid/scid mice. A total of 97% of the radioactivity was found to be intact tracer in plasma at 120 min post-injection by radio-TLC (Table 5) [84]. Similar observations were made for [*O-methyl*-^11^C]gefitinib by Zhang et al., who evaluated the metabolic stability in male fibrosarcoma-bearing mice at 60 min using radio-HPLC. In all the tissues collected (plasma, liver, kidney, and tumor), the intact tracer fraction was determined to be over 85% (Table 5) [83]. The metabolism of the [*O-methyl-* ^11^C]gefitinib in male ddY mice was slightly influenced by the P-gp and BCRP inhibitor elacridar. The percentage of intact tracer present in the brain was 88 ± 2% in control mice and 96 ± 1% in elacridar treated mice (5 mg/kg), while in plasma 93 ± 3% in control mice and 89 ± 5% in elacridar treated mice as determined by radio-HPLC 30 min post-injection (Table 5) [85]. Although not comparable due to the difference in the mass dose injected, these results are in discordance to the metabolite study in humans using [^14^C]gefitinib (50 mg), where only 9% of the radioactivity in plasma corresponded to intact [^14^C]gefitinib at one hour post-administration [86].

In the biodistribution of [^18^F]gefitinib in non-tumor-bearing SCID mice, the highest activity concentration was observed in the gallbladder, urinary bladder and parts of the intestine, with moderate accumulation in the kidneys and liver one hour post-injection. A similar pattern was observed in vervet monkeys, with the exception of the hepatobiliary and renal excretion, which was significantly slower [84]. Similar to [^18^F]gefitinib in mice, high liver and kidneys uptake was observed for [*O-methyl-* ^11^C]gefitinib in C3H/HeMsNrsf mice, which remained high, while radioactivity gradually accumulated in the small and large intestines during the 60 min post-injection [83].

The ability of [^18^F]gefitinib to determine EGFR status was evaluated in SCID mice xenografted with L858R mutated EGFR expressing H3255, L858R/T790M mutated EGFR expressing H1975, low wild-type EGFR expressing U87, and EGFR transfected U87-EGFR by PET. During a 90-min PET scan, no uptake could be observed in the tumors compared to surrounding muscle. The low uptake was confirmed by ex vivo biodistribution studies, where the tracer uptake was comparable in all tumors and lower than in blood and muscle (Table 6). To further elucidate the extent of the specific uptake, gefitinib was labeled with tritium and evaluated in vitro. Higher uptake of [^3^H]gefitinib was observed in L858R EGFR mutated H3255 than in wild-type EGFR expressing U87-EGFR. Furthermore, this uptake could be significantly reduced by pre-incubation with 20 µM gefitinib in H3255 cells in vitro. However, various experiments indicated that the amount of [^3^H]gefitinib bound to the EGFR was small compared to free or nonspecifically bound gefitinib [84].

[*O-methyl-*^11^C]gefitinib was evaluated in intact murine fibrosarcoma-bearing mice. The uptake was reported to be selective as it peaked in the fibrosarcomas between 30–60 min post-injection, while it decreased in blood and muscle. Furthermore, it was reported to be selective as treatment with the cold compound dose-dependently reduced the tumor uptake. Since the uptake could not be completely eliminated, it was assumed to be due to non-specific binding. The group reported that it was not possible to confirm the presence of EGFR in the murine fibrosarcomas by western blot, and therefore the uptake of [*O-methyl-*^11^C]gefitinib does not seem to be related to EGFR expression [83].

A known metastatic site of NSCLC expressing EGFR mutations is the brain. P-gp and BCRP are efflux transporters known to be functioning at the blood–brain barrier, and many TKIs are substrates of these [98]. It has long been thought that the resulting low brain concentration is the reason for the low efficacy of these TKIs in the treatment of brain metastases. Blocking these efflux transporters could increase the brain availability of the inhibitors. Therefore, brain penetration of [*O-methyl-*^11^C]gefitinib has been evaluated by modulating P-gp and BCRP efflux transporters. Co-injection with gefitinib (>50 mg/kg), cyclosporin A (P-gp modulator, 50 mg/kg), and elacridar (dual P-gp and BCRP modulator, >5 mg/kg) induced an increase in the brain uptake of [*O-methyl-*^11^C]gefitinib in mice 30 min post-injection. The relation between brain uptake of [*O-methyl-*^11^C]gefitinib and the efflux transporters P-gp and BCRP was furthermore confirmed when the brain uptake in wild-type FVB mice was found to be eight-fold lower than in BCRP and P-gp knock out mice at 60 min post-injection [85].

#### 2.3.2. Erlotinib

**Figure 12:**
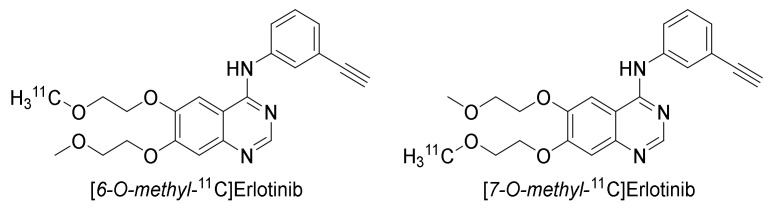
[*6-O-methyl*-^11^C]- and [*7-O-methyl*-^11^C]erlotinib.

Erlotinib is an orally available anilinoquinazoline that has a high affinity for EGFR-TK compared to other TK receptors [99]. Similar to gefitinib, erlotinib was evaluated as a treatment for pancreatic, ovarian, head and neck cancer, epithelial malignancies, gastrointestinal and genitourinary tracts, and gynecological malignancies [100]. Erlotinib has been labeled at two positions, [*6-O-methyl*-^11^C]- and [*7-O-methyl*-^11^C]erlotinib, and evaluated extensively both in preclinical and clinical studies (Figure 12). In case the position of the tracer could be verified in the referred publication by either specification of precursor or by the drawn structure, the position has been indicated hereafter. 

##### Preclinical Investigations

The metabolic stability of [*O-methyl*-^11^C]erlotinib was evaluated in two preclinical studies (Table 5). In female BALB/c nude CAnN.Cg-Foxn1nu/Crl mice, 95% intact tracer was observed in blood plasma at 25 min post-injection, as determined by radio-TLC (Table 5) [88]. In a study evaluating the influence of P-gp and BCRP on the organ distribution and excretion, the intact tracer concentration in plasma in female wild-type FVB mice was observed to be 80 ± 9%, while in the female BCRP/P-gp double knockout mice, it was 54 ± 12% at 25 min post-injection, as determined by radio-TLC (Table 5) [87].

The uptake of [*6-O-methyl*-^11^C]erlotinib in xenografted BALB/cA nude mice was correlated to the treatment sensitivity of the xenografted cell lines as determined by a MTT proliferation assay. In the Del19 EGFR mutation expressing HCC827 xenografts, a high radioactivity concentration could be detected, which was retained longer than in the EGFR wild-type expressing A549 and NCI358, where no significant uptake was observed. A549 and NCI358 xenografts were also found to be less sensitive to erlotinib treatment than HCC827 cells (Table 6) [95]. 

The ability of [*6-O-methyl-*^11^C]erlotinib to distinguish between EGFR expression status in vivo was evaluated in athymic nude-Foxn1^nu^ mice xenografted with SW620 (low wild-type EGFR), PC9 (Del19 EGFR mutation), HCC827 (Del19 EGFR mutation), U87(wild-type), and U87∆vIII (transfected with EGFRvIII). The influence of high molar activity versus low molar activity (10 mg/kg unlabeled erlotinib added to the tracer) was compared by PET. The highest uptake was observed in the Del19 EGFR mutation expressing HCC827, while the uptake in the other xenografts was comparable. However, when using the Simplified Reference Tissue Model kinetic modeling analysis, both of the Del19 EGFR mutation expressing cell lines, HCC827 and PC9, had the highest non-displaceable binding potential. Based on these results, the authors suggested that the selectivity of [*6-O-methyl-* ^11^C]erlotinib depended on the molecular and biophysical alterations caused by EGFR kinase domain mutations rather than EGFR expression level [94]. 

The ability of [*6-O-methyl-*^11^C]erlotinib to differentiate between primary and resistance mutations was assessed in Hsd:Athymic Nude-Fox1nu mice xenografted with four human NSCLC cell lines: QG56 (wild-type EGFR), HCC827 (De19 EGFR), H3255 (L858R), and H1975 (L858R/T790M) using µPET/CT. In all xenografts, except HCC827, a maximum uptake could be observed around 12 min, after which the uptake decreased. The tracer uptake in HCC827 was significantly higher than in H3255, H1975, and QG56 (Table 6). The uptake in the HCC827 xenografts could also be reduced to the same level as in H3255 xenografts by co-injection with erlotinib (6.7 mg/kg). The uptake in HCC827 xenografts was unexpectedly and notably different from that in H3255 xenografts. The in vitro cell proliferation of HCC827 and H3255 cells was significantly more sensitive to erlotinib treatment than QG56 and H1975. As the difference in EGFR expression (established by western blot) could not completely explain the lower uptake in H3255 in vivo, a difference in binding affinity of the EGFR TKI mutations was also considered a possible explanation [97]. 

Traxl et al., evaluated the ability of [*O-methyl*-^11^C]erlotinib to distinguish between treatment sensitive and treatment-resistant tumors using EGFR inhibitor-resistant sublines of the Del19 EGFR mutation expressing HCC827 cells and comparing them to the wild-type EGFR expressing A431. These new sublines were at least 100-fold more resistant to erlotinib treatment than the parental cell line, as determined by MTT assay. The tracer uptake was evaluated in tumor-bearing BALB/c nude CAnN.Cg-Foxn1nu/Crl mice. There was, however, no significant difference in the tumor total distribution volume (V_T_) between the erlotinib treatment-resistant cell lines and the parental HCC827 and A431. The total distribution volume for the tumor could be reduced in both the resistance mutated xenografts and the parental HCC827 xenografts by the co-injection of cold erlotinib (10 mg/kg). Based on the in vitro and in vivo studies, it was concluded that the tracer could not differentiate between treatment sensitive tumors and tumors that had developed treatment resistance [88].

##### Clinical Investigations

[^11^C]Erlotinib has been extensively evaluated in clinical trials. Two of these studies have reported the metabolism of the tracer (Table 5). In a study by Bahce et al., the metabolic stability of [*7-O-methyl*- ^11^C]erlotinib was assessed by radio-HPLC in NSCLC patients with and without EGFR activating mutations. The intact tracer percentage in the groups was 43 ± 7% and 54 ± 2%, respectively, while the polar radioactive metabolites made up 33 ± 17% and 31 ± 21% of the plasma radioactivity 60 min following the injection. The non-polar metabolite fraction was reported to be too small to produce reliable quantification on the radio-HPLC, therefore, solid-phase extraction analysis of the metabolism was considered sufficient [89]. Bauer et al., however, published contradicting observations when evaluating the effect of rifampicin on the distribution of [*6-O-methyl*- ^11^C]erlotinib to the liver in humans. Solid-phase extraction yielded less than 10% polar metabolites at 20 min post-injection. The amount of intact tracer was therefore determined to be 98.0 ± 0.5% in healthy volunteers 20 min post-injection. The percentage of intact tracer was, however, not determined in the same way as Bahce et al.: after solid-phase extraction of the plasma fraction, the percentage of intact tracer was not determined by means of radio-TLC or radio-HPLC, therefore the results are not comparable [90]. In the metabolism evaluation of [^14^C]erlotinib (100 mg oral dose), the main component circulating in plasma was intact [^14^C]erlotinib (82.8 ± 4.6%) in male volunteers at 2 h post-ingestion [101].

Similar to the preclinical evaluation of [^18^F/*O-methyl-*^11^C]gefitinib, a multi-species radiation dosimetry evaluation of [*O-methyl*-^11^C]erlotinib revealed the liver, gallbladder, and the gastrointestinal tract to be discernable from the background 30 min post-injection. The liver TAC was comparable between species; however, the liver kinetics in the human participant was slower than in pig, mice, or monkeys. The liver was identified as the critical organ in three of four species [102].

**Figure 13:**
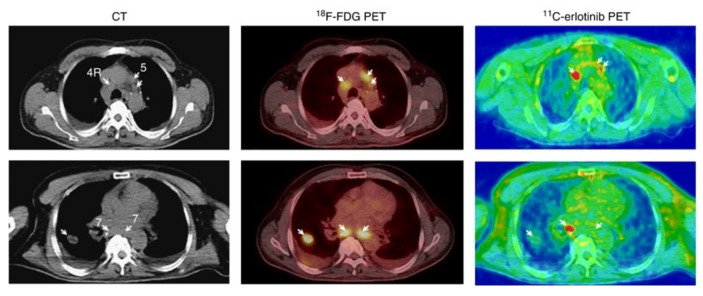
The observed variation in [*6-O-methyl-*^11^C]erlotinib accumulation in different NSCLC tumor foci in the same patient. Adapted from [103], with permission from Springer Nature.

The ability of [*6-O-methyl-*^11^C]erlotinib to identify the patients most likely to benefit from erlotinib treatment was evaluated in a clinical study where 13 patients with NSCLC received a 90 min PET/CT scan to determine the accumulation of [*6-O-methyl-*^11^C]erlotinib before the initiation of erlotinib treatment. Twelve weeks after initiation, five patients exhibited stable disease, of which three displayed [*6-O-methyl*-^11^C]erlotinib hotspots. A variation in the tracer accumulation in different tumors in the same patient was observed (Figure 13). It was suggested that not all tumors or metastases would express the same driving mutations or statuses. Unfortunately, the EGFR mutational status was not confirmed in the study to further investigate this option [103]. The same authors were also involved a case study where the uptake of [*6-O-methyl-*^11^C]erlotinib corresponded to metastatic lesions in the brain of a NSCLC patient with an EGFR Del19 mutated lung tumor [104].

Bahce et al., quantified the tumor uptake and correlated uptake to the EGFR mutational status of the tumors. Ten patients with NSCLC were enrolled in a study, five lacking and five expressing an activating EGFR Del19 mutation. The volume of distribution of [*7-O-methyl*-^11^C]erlotinib was significantly larger in all subjects in the group with Del19 mutation-positive tumors compared to the group with wild-type EGFR or exon 20 mutations. Three of the five patients with Del19 mutated tumors had a partial response to erlotinib treatment. As some of the patients in the study had received TKIs prior to the study, resistance mutations might have been present at the time of the study. This could in turn have also impacted the tracer uptake [89]. 

In the follow-up clinical study, the capability of the tracer to image tumors expressing EGFR activating mutations in patients on or off erlotinib treatment was evaluated. A decrease in tumor uptake was observed during erlotinib treatment. Similar to preclinical observations [87], it was observed that the intact tracer fraction in arterial plasma samples and the arterial blood activity was higher in patients on erlotinib treatment. It was established that T/B ratios at 40–60 min correlated well with V_T_ values, while SUVs did not [105].

##### Brain Uptake of [O-methyl-^11^C]erlotinib

As the brain is a known metastatic site of NSCLC expressing EGFR mutations and many TKIs are substrates of efflux transporters functioning at the blood–brain barrier, the influence of BCRP and P-gp on the brain penetration of [*O-methyl*-^11^C]erlotinib has been evaluated in various studies. 

The brain uptake of [*6-O-methyl*-^11^C]erlotinib was low in wild-type mice compared to mice where both the P-gp and the BCRP were knocked out. Knocking out either the P-gp or the BCRP resulted in a moderate but not significant increase in brain accumulation. A pre-injection of the dual inhibitor elacridar (10 mg/kg, 20 min prior) significantly increased the brain AUC in wild-type mice to almost the same level as in the double (P-gp/BCRP) knockout mice. The uptake in the brain was also increased in both the wild-type mice and the double (P-gp/BCRP) knockout mice when receiving a pharmacological dose of erlotinib (10 mg/kg) [87]. 

The influence of eight compounds, with known in vitro P-gp/BCRP inhibitory properties, on the brain uptake of [*6-O-methyl*-^11^C]erlotinib was evaluated in vivo. The total distribution volume V_T_ for [*6-O-methyl*-^11^C]erlotinib was increased when tariquidar, erlotinib, imatinib, lapatinib, and cyclosporine A were administered as a continuous i.v. infusion during the PET scan, but not for nilotinib, pazopanib, and osimertinib. However, the increase was lower than in P-gp/BCRP knockout mice, so only partial inhibition seemed to occur. Increased blood radioactivity concentration could also be observed for some of the evaluated compounds, possibly due to inhibition of hepatobiliary excretion [106]. 

It is well-known that P-gp and BCRP expression levels at the blood–brain barrier are markedly different between mice and humans, so the effect of dual ABCB1/ABCG2 blocking in non-human primates was also assessed using [*6-O-methyl*-^11^C]erlotinib. Similar to previous studies, the brain V_T_ of [*6-O-methyl*-^11^C]erlotinib could be increased in male Papi Anubis baboons when administered with elacridar by continuous intravenous infusion maintained for the duration of the PET scan. Elacridar did not seem to influence the plasma pharmacokinetics of [*6-O-methyl*-^11^C]erlotinib, indicating the increase to be solely due to transporter inhibition at the blood–brain barrier. An increase in brain V_T_ could also be achieved when erlotinib was co-infused during the PET scan. This, however, did increase the [6-*O-methyl*-^11^C]erlotinib plasma levels [107]. 

Since elacridar has poor water solubility, the formulation of an i.v. administration for humans is extremely challenging. In contrast, tariquidar is more water-soluble and therefore suitable for i.v. administration. The compound mainly inhibits P-gp (not BCRP) at clinically tolerable doses, and has been shown to increase the brain penetration of (R)-[^11^C]verapamil and [^11^C]N-desmethyl-loperamide in healthy human volunteers. Since P-gp and BCRP have been shown to work in concert, a significant increase in brain uptake is only expected when both are blocked simultaneously. Therefore, the influence of tariquidar and/or erlotinib was evaluated in FVB mice and nonhuman primates (Macaca mulatta). An increase in brain exposure (AUC_brain_) and blood–brain barrier penetration (V_T_ and k) of [*6-O-methyl*-^11^C]erlotinib was observed in both mice and nonhuman primates when administered with both erlotinib and tariquidar. Infusion of each drug on its own was observed to have less influence. Comparing the AUC for brain to that of muscle suggested complete inhibition of ABC transporter-mediated efflux of [6-*O-methyl*-^11^C]erlotinib. Increased plasma concentrations of the tracer could be observed in both mice and nonhuman primates, but kinetic modeling showed that this was not the sole reason for the increased brain exposure [108]. 

The effect of the dual P-gp/BCRP inhibitor elacridar on the brain uptake of [*O-methyl*-^11^C]erlotinib was studied in humans and compared to that in mice. The brain uptake of cold erlotinib could be increased in FVB mice by administering elacridar (0–50 mg/kg administered by oral gavage). A marked difference in brain uptake of the cold erlotinib could also be observed between FVB mice and P-gp/BCRP knockout mice. Similarly, the brain uptake of [*O-methyl*-^11^C]erlotinib was 2.6-fold higher in knockout mice than in FVB mice when compared by PET imaging. The influence of elacridar on the brain uptake of [*O-methyl*-^11^C]erlotinib was evaluated in four patients with gastrointestinal stromal tumors or colorectal cancer. However, contrary to the preclinical results, no effect on the [*O-methyl*-^11^C]erlotinib V_T_ was observed when elacridar (1000 mg 8–12 h before scan) was administered orally. The discrepancy between species was thought to be due to the difference in ABCG2 expression and elacridar’s potency for its inhibition [109]. 

The influence of tariquidar and different oral doses of erlotinib (300, 650, or 1000 mg) on the brain uptake of [*6-O-methyl*-^11^C]erlotinib was also studied in healthy male volunteers. The erlotinib oral intake was observed to slightly influence the fraction of intact tracer in plasma. Though not to the same extent as in the non-human primate study [108], the [*6-O-methyl-*^11^C]erlotinib brain V_T_ was increased by 27% when 1000 mg erlotinib was administered orally three hours before the PET scan. The AUC, however, was increased by 94%. Tariquidar was found to increase the brain uptake of [*6-O-methyl*-^11^C]erlotinib solely in one volunteer who was a single-nucleotide polymorphism carrier. These carriers have reduced ABCG2 transport activity at the BBB, leading to higher increases in brain distribution [110].

#### 2.3.3. Afatinib

**Figure 14:**
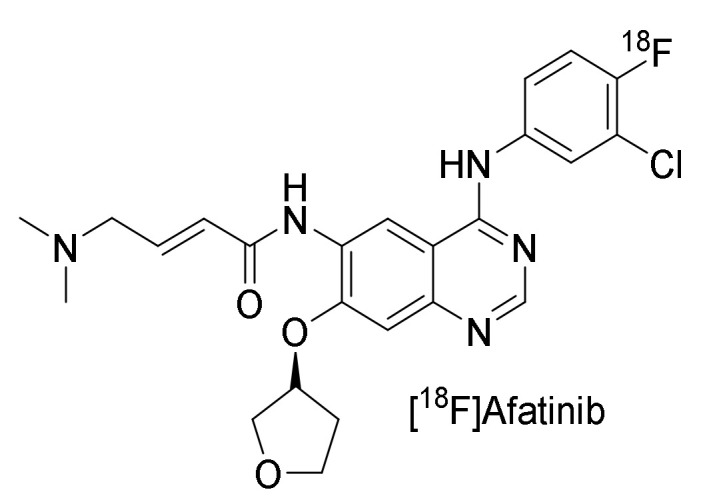
[^18^F]Afatinib.

Treatment resistance in the form of the EGFR mutation T790M and other small in-frame insertions in both EGFR and HER2 was observed following treatment with reversible binding tyrosine kinase inhibitors. Afatinib was developed with a Michael acceptor moiety enabling irreversible binding to Cys 773 of the EGFR TK, Cys 805 of HER2, and Cys 803 of ErbB-4 [20,111]. ^18^F-labeled [^18^F]afatinib (Figure 14) has been both preclinically and clinically evaluated [91,92,96].

##### Preclinical Investigation

Afatinib was labeled with fluorine-18 on the aniline ring. The metabolic stability was evaluated in BALB/c mice, 83 ± 1% of the radioactivity in plasma was intact tracer while 11 ± 2% of the radioactivity was attributed to apolar metabolites at 45 min post-injection (Table 5). The biodistribution was evaluated in tumor-bearing nude mice, which revealed a rapid and high uptake in kidneys and liver [91].

The tumor-targeting potential of [^18^F]afatinib was evaluated in nude mice xenografted with wild-type EGFR expressing A549, Del19 EGFR mutation expressing HCC827, and L858R/T790M EGFR resistance mutated H1975 xenografted by both PET and ex vivo biodistribution. In all three xenografts, the radioactive concentration peaked after 5 min post-injection and was retained for 120 min (Table 6). Co-administration of 100 ng of afatinib reduced the uptake in HCC827 xenografts to background level. Although a therapeutic effect has been observed in H1975 tumors upon treatment with afatinib, the uptake in the xenografts did not differ from uptake in muscle, which was used as reference tissue [91,96].

The tumor-targeting potential of [^18^F]afatinib was compared to that of [*7-O-methyl*-^11^C]erlotinib in nude mice xenografted with A549, HCC827, and H1975 by PET imaging. The main observations were that [*7-O-methyl*-^11^C]erlotinib cleared slower from the blood than [^18^F]afatinib and that the radioactive concentration peaked at 10 min post injection for [^18^F]afatinib, while it peaked at 25 min post-injection for [*7-O-methyl*-^11^C]erlotinib in HCC827 tumors. Despite the significant differences in maximum tumor uptake (Table 6), the tumor-to-background ratio at the end of the imaging was the same. The uptake in resistance mutated H1975 xenografts was comparable to the background for both tracers. While there was no significant difference between uptake of [*7-O-methyl*-^11^C]erlotinib in wild-type EGFR expressing A549 tumors and background, the A549 tumor uptake for [^18^F]afatinib was significantly higher than the background [96].

##### Clinical Investigations

A feasibility study evaluating the PET quantification of [^18^F]afatinib in NSCLC patients was published by van de Stadt et al., in 2020. The metabolic stability was evaluated in ten NSCLC patients, of which seven possessed tumors expressing primary EGFR mutations. In humans, the intact tracer fraction in plasma as determined by HPLC, decreased from 70% at 5 min post-injection to 30% at 75 min post-injection (Table 5). The best kinetic model for tumor uptake was found to be an irreversible two-tissue compartment model and a metabolite corrected sampler-based input function. The preferred simplified uptake parameter was tumor-to-blood ratio over a 60- to 90-min time interval [92].

#### 2.3.4. Osimertinib

Osimertinib was developed as a third-generation covalently binding tyrosine kinase inhibitor that structurally and pharmacologically differs from previously described EGFR TK inhibitors. It was developed to inhibit the T790M resistance mutation while sparing the wild-type EGFR to reduce EGFR TKI related side-effects. The inhibitor is also able to inhibit primary EGFR sensitizing mutations [112].

##### Preclinical Investigation

**Figure 15:**
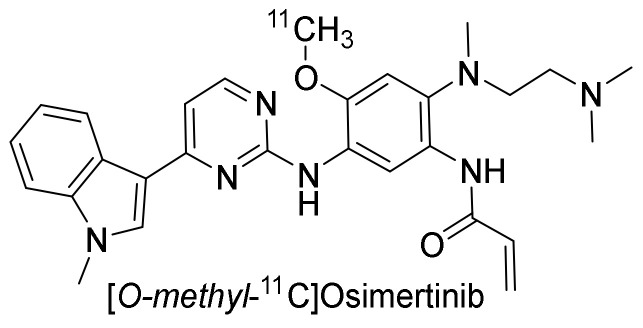
[*O-methyl-*^11^C]Osimertinib.

**Figure 16:**
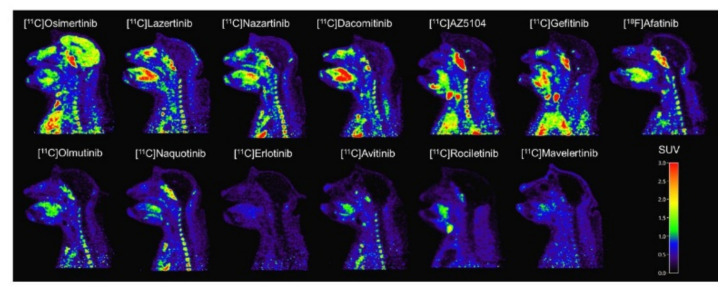
The brain uptake of [*O-methyl-*^11^C]osimertinib compared to other radiolabeled TKIs. Adapted from [113], with permission from AACR.

[*O-methyl-*^11^C]osimertinib (Figure 15) was developed for a PET study comparing the blood–brain barrier penetration to that of [*O-methyl-*^11^C]gefitinib, [*O-methyl-*^11^C]rociletinib, and the main metabolite [112] of osimertinib, [*O-methyl-*^11^C]AZ5104, in cynomolgus monkeys. The uptake of [*O-methyl-*^11^C]osimertinib in the brain was fast and plateaued within 10 min post-injection. The highest radioactivity concentration at 10 min was observed with [*O-methyl-*^11^C]osimertinib (1.29 ± 0.42%ID), while uptake of [*O-methyl-*^11^C]gefitinib (0.11%ID), [*O-methyl-*^11^C]rociletinib (0.023 %ID), and [*O-methyl-*^11^C]AZ5104 (0.17%ID) was much lower [98]. The comparison was further extended to nine other TKIs (Figure 16). The following were radiolabeled and compared to [*O-methyl-*^11^C]osimertinib: [*O-methyl-*^11^C]lazertinib, [*dimethylamine*-^11^C]nazartinib, [*O-methyl-*^11^C]dacomitinib, [^18^F]afatinib, [*methylpiperazine-*^11^C]olmutinib, [*methylpiperazine-*^11^C]naquotinib, [*7-O-methyl-*^11^C]erlotinib, [*methylpiperazine-*^11^C]avitinib, [*O-methyl-*^11^C]rociletinib, and [*O-methyl-*^11^C]mavelertinib. The maximum radioactivity concentration (C_max_) in the brain for [*O-methyl-*^11^C]osimertinib was 1.49 ± 0.39%ID at 100 min. None of the other 12 TKIs surpassed a C_max_ of 0.56%ID. Considering the brain of the macaques accounts for less than 1% of their body weight, the high C_max_ for [*O-methyl-*^11^C]osimertinib was considered to suggest preferential distribution of radioactivity to the brain. The area under the brain radioactivity concentration–time curve corrected for the radioactivity in the cerebral blood also suggested the preference for brain over blood [113]. 

##### Clinical Investigations

The blood–brain barrier penetration, distribution, and brain exposure of [*O-methyl-*^11^C]osimertinib was investigated in healthy human subjects. The stability of [*O-methyl-*^11^C]osimertinib in plasma was 73 ± 8% at 30 min as assessed by HPLC. As in the cynomolgus monkey study, there was a rapid uptake of radioactivity in the brain. The maximum uptake in the brain was achieved at 13 min (2.2 ± 0.2%ID) [93].

#### 2.3.5. Toward Standardizing Evaluation of EGFR TKI PET Tracers

It is important to standardize the evaluation of tracers to enable comparison between tracers. Though structurally related, comparing the metabolism of the ML-series of PET tracers with the group of clinically approved TKI-based PET tracers is challenging. The metabolism of the PET tracers in the ML-series was studied by extracting blood with ether, followed by the analysis of the extracted fraction using radio-TLC. The metabolism of the PET tracers based on clinically approved TKIs has often been studied by separating the plasma from the blood and analyzing the plasma for the percentage of intact tracer by analytical methods such as radio-TLC or -HPLC (Table 5). While this has become the new standard, we have lost the information on the fraction of intact tracer present in plasma compared to the radioactivity concentration in the rest of the blood.

PET tracer uptake has mainly been correlated to the EGFR mutational status of the cell lines. Cell lines that inherently express mutated EGFR are H3255 (L858R), HCC827 (Del19), PC9 (Del19), and H1975 (L858R/T790M). A few studies have included other cell lines either as negative control cell lines, or to demonstrate that no EGFR uptake difference could be detected in cell lines expressing different levels of EGFR. The most commonly used mutation expressing cell line is HCC827, which expresses a Del19 mutation, as can be seen in the Table 6.

## 3. Discussion

In this review, we summarized a few key observations for small molecules developed as PET tracers for the imaging of EGFR-TK. The majority of studies described in this review have evaluated tracers by the ability to differentiate tumors according to either the expression level of EGFR/pEGFR or mutational status. In general, between studies, very few cell lines have been repeatedly used for either purpose, making a comparison of results challenging. However, most of the recent studies have focused on the tracer’s ability to differentiate according to mutational status. As shown in Table 7, the selectivity between purified wild-type EGFR and mutated EGFR kinase is not always marked. This can lead to skewed results if the expression level of mutated or non-mutated EGFR is not equal between cell lines and can be one of the reasons why a difference in tumor uptake is not observed.

The most common cell line used as an indicator for successful EGFR targeting is the Del19 EGFR mutation expressing HCC827. The uptake in this cell line is subsequently compared to wild-type EGFR or no EGFR expressing cell lines. Very seldomly has the uptake in the Del19 EGFR mutation expressing PC9 been comparable to HCC827, indicating that solely the existence of the mutation is not enough. Various studies have tried to explain the difference by comparing the EGFR or the pEGFR expression level in cells in vitro. The primary mutation L858R expressing H3255 has neither demonstrated the selectivity of any PET tracers in vivo. Similar to PC9, the cell line has shown sensitivity to treatment with TKI, however, the uptake has not been comparable to that of HCC827. It therefore raises the question of whether the expression level of the mutated EGFR is too low in these cell lines compared to the off-target interaction of the anilinoquinazolines.

An additional challenge in the development of EGFR-TK PET tracers is that many of the tracers are multi-kinase inhibitors due to the similarity between the kinases. The EGFR receptor family is closely related, with the tyrosine kinase domain homology between EGFR and HER2, HER3, and HER4 being 82, 59, and 79%, respectively [117]. Although the affinity of the tracers for various kinases may vary, information on the relative expression level of these kinases in the cell lines used to evaluate the PET tracers might not always exist or be readily available. Similarly to the situation described above, if the tumor uptake is compared in two different xenografts, but the expected “negative” cell line expresses a large amount of HER2, for example, the difference might not be as marked as expected.

Notably, several studies have included blocking experiments to demonstrate the specificity of the tracer. A trend has been to do so by self-blocking. This, however, is not a favorable approach as any affinity for another kinase will not be illustrated by self-blocking as the cold compound will also block these off-target receptors equally well. Therefore, the use of another TKI to block the EGFR is recommended to limit the risk of occupying the same off-target kinases the compound might show affinity for.

**Figure 17:**
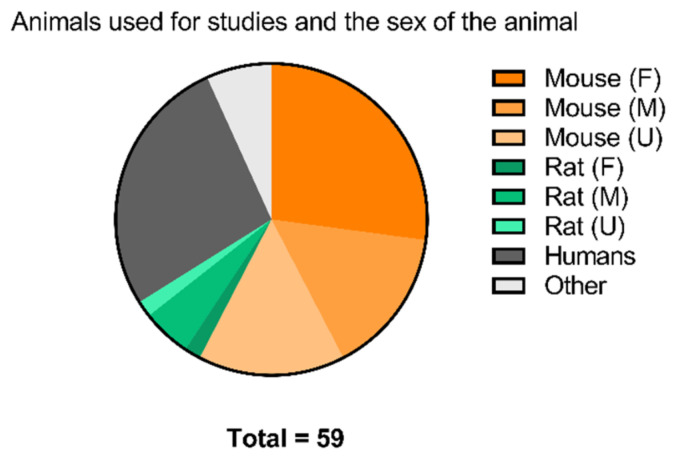
Animals used for EGFR TKI PET studies and the reported sex of the animal. Female (F), Male (M), and Unknown (U).

The most common species used for preclinical evaluation of small molecule PET tracers are rats and mice. Tumor development has been shown to be more challenging in rats than mice, but when it comes to blood sampling, rats offer the possibility to carry out more frequent or larger blood sampling. The biodistribution evaluation of morpholino-[^124^I]-IPQA demonstrated the difference that could result from the choice of animal. Both a difference in uptake as well as tumor-to-blood and -muscle ratios could be seen in tumors grown in rats and mice. Most of the tracers presented in this review have been evaluated in mice (Figure 17). In the ex vivo biodistribution of [^125^I]I-CO1686, a large difference in the radioactivity concentration in the liver and small and large intestine could be detected between male ddY mice and female BALB/C nu/nu mice. Whether this was due to the difference in strain or the sex of the mice was not further discussed in the publication.

In a large percentage of studies, the sex of the study subjects has not been reported (Figure 17). As it has been shown that the sex of the subject may influence, for example, the pharmacokinetics, metabolism, and bioavailability of xenobiotics, it is beneficial for the studies to take this into account [118]. PD153035, gefitinib, erlotinib, and osimertinib have all been indicated to be metabolized to a substantial degree by P450 cytochromes, especially CYP3A4 [37,101,112,119]. In humans, the P450 cytochrome CYP3A4 is expressed at a higher protein and mRNA level in women than in men [120], and e.g., rats have sex-specific cytochrome P450 in the liver [121]. Furthermore, NSCLC adenocarcinomas expressing EGFR mutations are statistically more prevalent in female non-smokers [122]. When the plasma protein binding of [^14^C]gefitinib was evaluated in different species, there was no indication of the influence of sex, except in mice. The proportion of free drug was markedly lower in males than in females. A slight difference in the recovered dose following a 5 mg/kg oral or intravenous injection of [^14^C]gefitinib in female and male rats could also be observed (102.1 ± 2.9% vs. 92.9 ± 1.5% recovered dose found in feces in orally administered and 98.3 ± 1.4% vs. 94.2 ± 1.1% recovered dose in i.v. injected rats). Unfortunately, only male volunteers were included in the study on the metabolism of [^14^C]gefitinib in humans, so it was not determined whether there was a sex difference in human metabolism [86]. Following oral administration of [^14^C]osimeritinib, it was observed that the distribution in female albino rats was similar to that of males, though the concentration of radioactivity in the blood was generally higher in female than in male rats [112].

## 4. Conclusions

In conclusion, EGFR TKI PET tracers started as tools for quantifying and locating tissues expressing high concentrations of EGFR, but very soon became tools for specifically locating tumors expressing mutated EGFR receptors. A lack of comparability both in evaluation techniques and results combined with uncertainty in evaluation approaches hinders the development of these tracers. The current evaluation trend compares tumor uptake to treatment sensitivity or mutational status of cell lines. However, the treatment sensitivity is usually evaluated by different inhibition assays such as the comparison of the phosphorylation of the (purified) kinase and the influence on metabolic viability or growth of cells, which does not guarantee a similar response in the corresponding xenograft upon treatment with the compound. The mutational status also does not guarantee a high uptake as it depends on the expression level of the mutated kinase and the specificity for said kinase. Affinity for other kinases might skew results, resulting in misleading conclusions. A better understanding of the animal models is thus needed to guarantee reproducible and comparable results, which can then be used to drive the development of these tracers.

## Figures and Tables

**Table 1:** Xenografts used for the assessment of the tumor-targeting potential of [*O-methyl*-^11^C]PD153035.

Cell Line	Disease	Tissue	EGFR Status [41]	Other Mutations [41]	Maximum Uptake (Determined By)
SH-SY5Y	Neuroblastoma	Bone marrow	Wild-type EGFR	ALK	0.23–0.33%ID/mL at 8 min * (PET) [36]
MDA-MB-231	Adenocarcinoma	Mammary gland/breast; pleural effusion	Wild-type EGFR	CDKN2A, BRAF, KRAS, TERT, TP53	0.54 ± 0.10%ID/g at 10 min (ex vivo), T/M 0.84 [38]
MDA-MB-468	Adenocarcinoma	Mammary gland/breast; pleural effusion	Wild-type EGFR (amplification)	PTEN, RB1, TP53	1.39 ± 0.28%ID/g at 10 min (ex vivo), T/M 1.81 [38]
A549	Carcinoma	Lung	Wild-type EGFR	KRAS, STK11, TP53	0.89 ± 0.13%ID/g at 10 min (ex vivo), T/M 1.32 [38] Unknown (PET) [42]
HCC827	Adenocarcinoma	Lung	Del19	TP53	Unknown (PET) [42]
H1975	Adenocarcinoma; NSCLC	Lung	L858R/T790M	PIK3CA, TP53	Unknown (PET) [42]
A431	Epidermoid carcinoma	Skin/epidermis	EGFR-PPARGC1A fusion	TP53	0.3%ID/g at 2–3 min (PET) [37]
PC9	Adenocarcinoma	Lung	Del19	TP53	Unknown (PET) [42]

* Approximate value from the data presented in [36].

**Table 2:** Metabolism and maximum uptake of the ML-compounds in A431 tumors.

Tracer	Animal	Metabolism	Method	Maximum Uptake in A431 Xenografts
1	BALB cBy nu/nu male mice	Extractable fraction in blood: 16% Intact tracer in the fraction: 75% at 20 min	Radio-TLC [48]	Ex vivo: 1.34 ± 0.79%ID/g T/B 1.62 ± 0.68 at 60 min [48]
2	BALB cBy nu/nu male mice	Extractable fraction in blood: 36% Intact tracer in the fraction: 56% at 10 min	Radio-TLC [48]	Ex vivo: 2.90 ± 1.67%ID/g T/B 0.89 ± 0.63 at 5 min In vivo: T/B ratio of 0.55, T/M ratio of 0.83 at 10 min [48]
3	WAG rnu/rnu male rats	Extractable fraction in blood: 32% Intact tracer in the fraction: 90% at 15 min Extractable fraction in blood: 17% Intact tracer in the fraction: 75% at 60 min	Radio-TLC [49]	Ex vivo*:* 0.09 ± 0.039%ID/g T/B 0.54 ± 0.04 at 15 min In vivo*:* T/B 2.86, T/M 4.61 at 120 min [49]
	Nude Hsd:RH-rnu/rnu male rats	Intact tracer in blood: 29% at 15 min * Intact tracer in blood: 13% at 60 min *	Radio-TLC [50]	ND
4	Nude Hsd:RH-rnu/rnu male rats	Intact tracer in blood: 40% at 15 min * Intact tracer in blood: 38% at 60 min *	Radio-TLC [50]	ND

* Reviewer estimated values, ND = not determined.

**Table 3:** IC_50_ values for PD153035 and **1–6** based on either autophosphorylation (AP) in EGFR tyrosine kinase from cell lysate or intact A431 cells, or by measuring cell growth inhibition in intact A431 cells.

	PD153035 [48]	1 [48]	2 [48]	3 [50]	4 [50]	5 [51]	6 [51]
Lysate EGFR TK AP	0.19 nM	75 nM	0.21 nM	0.04 nM	0.11 nM	ND	ND
A431 EGFR AP	14.6 nM	3230 nM	3.8 nM	6.7–20 nM	4–10 nM	3 ± 2 nM	8 ± 2.5 nM
A431 EGFR AP, 8 h *	ND	ND	ND	6.7–20 nM	10–50 nM	15 ± 7 nM	20 ± 5 nM
A431 cell growth	~2.9 μM	~10 μM	4.5 μM	ND	ND	ND	ND

* Concentration required to suppress kinase activity up to 8 h after the removal of the inhibitor, ND = not determined.

**Table 4:** Cell lines used and maximum tracer uptake for EGFR TKI PET tracers not based on clinically approved EGFR TKIs.

Cell Line	Tissue	Disease	EGFR Status	Other Mutations [41]	Tracer	Maximum Tracer Uptake
U87 (U87MG)	Brain	Likely glioblastoma	Low amount of wild-type EGFR	IDH1, NF1, PTEN, TERT, TP53	[^18^F]ML04	Transfected with EGFR: 0.92 ± 0.03%ID/g, T/B 6.97 ± 0.52, T/M 5.11 ± 0.29 at 3h [52]
					ML04-PEG_4_-[^18^F]F	Transfected with EGFR: Not specified [53]
					[^11^C]ML04-PEG_4_-OH	Transfected with EGFR: Not specified [53]
					[^124^I]IPQA-PEG_4_-OH	Transfected with EGFR: Not specified [53]
U138MG	Brain	Glioblastoma	EGFR-negative	CDKN2A, IDH1, PTEN, TP53	[^18^F]ML04	0.68%ID/g at 3h [52]
					ML04-PEG_4_-[^18^F]F	Not specified [53]
					[^11^C]ML04-PEG_4_-OH	Not specified [53]
					[^124^I]IPQA-PEG_4_-OH	Not specified [53]
H441	Lung	Papillary adenocarcinoma	Wild-type EGFR	KRAS, TP53	IPQA-PEG_6_-[^18^F]F	1.59 ± 0.06 %ID/g, T/M 1.47 ± 0.09 at 120 min [54]
					[^77^Br]Br-CO1686	3.71 ± 0.13 %ID/g at 60 min [55]
					[^125^I]I-CO1686	0.44 ± 0.06 %ID/g at 60 min [55]
H1975	Lung	Adenocarcinoma; NSCLC	L858R/T790M	PIK3CA, TP53	IPQA-PEG_6_-[^18^F]F	1.17 ± 0.18%ID/g, T/M 1.05 ± 0.10 at 120 min [54]
					[^18^F]MPG	3.93 ± 0.44%ID/g at 60 min, T/M ratio not specified [56]
					[^18^F]APP-1	T/M 2.95 at 180 min [57]
					[^18^F]F-IRS	1.71 ± 0.18%ID/g at 120 min [58]
					6-O-[^18^F]FEE	SUV of 0.5 at 60 min [59]
					[^125^I]I-CO1686	1.77 ± 0.43%ID/g[60], 0.68 ± 0.11%ID/g at 60 min [55]
					[^77^Br]Br-CO1686	4.51 ± 0.17%ID/g at 60 min [55]
					[^125^I]I-osimertinib	1.97 ± 0.30%ID/g at 4h [61]
					[^77^Br]Br-osimertinib	1.96 ± 0.33%ID/g at 4h [61]
H3255	Lung	Adenocarcinoma	L858R	TP53	IPQA-PEG_6_-[^18^F]F	2.34 ± 0.13%ID/g, T/M 2.08 ± 0.19 at 120 min [54]
					[^18^F]APP-1	3.80 ± 0.88%ID/g, T/M 13.37± 4.02 at 180 min [57]
					[^125^I]I-CO1686	1.63 ± 0.23%ID/g at 60 min [60]
					[^125^I]I- osimertinib	2.93 ± 0.11%ID/g at 4h [61]
					[^77^Br]Br-osimertinib	3.42 ± 0.05%ID/g at 4h [61]
PC14 *	Lung; Lymph node	Adenocarcinoma	Del19	TP53	IPQA-PEG_6_-[^18^F]F	0.99 ± 0.18%ID/g, T/M 0.9 ± 0.11 at 120 min [54]
K562	Bone marrow	Chronic myelogenous leukemia	Low amount of wild-type EGFR	BCR-ABL1, TP53	Morpholino-[^124^I]IPQA	rats: 0.20 ± 0.03%ID/g, T/M 1.25 at 60 min [62] mice: 0.43 ± 0.02%ID/g, T/M 1.79 at 69 min [62]
A431	Skin/ epidermis	Epidermoid carcinoma	EGFR-PPARGC1A fusion	TP53	Morpholino-[^124^I]IPQA	rats: 0.72 ± 0.12%ID/g, T/M 4.52 at 60 min [62] mice: 1.32 ± 0.26%ID/g, T/M 5.8 at 69 min [62]
H1299	Lung; lymph node	Carcinoma; NSCLC	Wild-type EGFR	TP53, NRAS	Morpholino-[^131^I]IPQA	Transfected with L858R: 0.28 ± 0.00%ID/g, Del19: 0.30 ± 0.00%ID/g, EGFR: 0.35 ± 0.00%ID/g, Vector: 0.22 ± 0.00%ID/g at 60 min [63]
HCC827	Lung	Adenocarcinoma	Del19	TP53	[^18^F]MPG	7.22 ± 0.28%ID/g, T/M 5.56 at 60 min [56]
					[^18^F]F-FEA-erlotinib	0.70 ± 0.37%ID/g at 15 min, T/M 3.19 ± 0.5 at 60 min [64]
					6-O-[^18^F]FEE	SUV of 1.0 at 60 min [59]
					[^18^F]F-IRS	4.27 ± 0.15 %ID/g at 120 min [58]
H520	Lung	Squamous cell carcinoma	EGFR-negative	ATM, CDKN2A, TP53	[^18^F]MPG [^18^F]F-IRS	3.59 ± 0.93%ID/g at 60 min, T/M ratio not specified [56] 1.62 ± 0.08%ID/g at 120 min [58]
H358	Lung /bronchiole	bronchioalveolar carcinoma: NSCLC	Wild-type EGFR	TP53, KRAS	[^18^F]MPG [^18^F]F-IRS	4.11 ± 0.46%ID/g at 60 min, T/M ratio not specified [56] 1.68 ± 0.29%ID/g at 120 min [58]
QG56	Lung	Squamous cell carcinoma	Wild-type EGFR	TP53	6-O-[^18^F]FEE	SUV of 0.3 at 60 min [59]
S180	Murine	Sarcoma			[^18^F]icotinib derivative 1a	1.61 ± 0.33%ID/g, T/M 1.45 at 30 min [65]
					[^18^F]icotinib derivative 1b	4.70 ± 0.23%ID/g, T/M 2.3 at 30 min [65]
					[^18^F]icotinib derivative 1c	3.06 ± 0.22%ID/g, T/M 1.11 at 30 min [65]
A549	Lung	Carcinoma	Wild-type EGFR	KRAS, STK11, TP53	[^18^F]icotinib derivative 1d	0.90 ± 0.24%ID/g at 90 min [66]

* PC14 short tandem repeat DNA profile analysis showeed this cell line to be identical to PC-9.

**Table 5:** Metabolism of tracers based on clinically approved TKIs.

Tracer	Animal	Metabolism	Method
[^18^F]Gefitinib	Scid/Scid mice	Intact tracer in plasma: >97% at 120 min	Radio-TLC [84]
[^11^C]Gefitinib	ddY mice	Intact tracer in plasma: 93 ± 3.3% at 30 min	Radio-HPLC [85]
	C3H/HeMsNrsf mice	86.2 ±1.5% at 60 min	Radio-HPLC [83]
[^11^C]Erlotinib	FVB/N wild-type mice Abcb1a/b(-/-)Abcg2(-/-) mice	Intact tracer in plasma: 80 ± 9% at 25 min 54 ± 12% at 25 min	Radio-TLC [87]
	BALB/c nude CAnN.Cg-Foxn1nu/Crl	Intact tracer in plasma: >95% at 25 min	Radio-TLC [88]
	NSCLC patients	Intact tracer in plasma: 54 ± 2% at 60 min 43 ± 7% at 60 min	Radio-HPLC [89]
	Healthy human volunteers	Intact tracer in plasma: 96.4 ± 1.3% at 40 min	Solid-phase extraction [90]
[^18^F]Afatinib	BALB/c mice	Intact tracer in plasma: 83.3 ± 1.3% at 45 min	Radio-HPLC [91]
	NSCLC patients	Intact tracer in plasma: 30% at 75 min	Radio-HPLC [92]
[^11^C]Osimertinib	Healthy human volunteers	Intact tracer in plasma: 73 ± 8% at 30 min	Radio-HPLC [93]

**Table 6:** Overview of tumor uptake and IC_50_ values of isotopologue labelled clinically approved TKI drugs.

	U87	A549	H3255	PC9/PC14	HCC827	H1975	Other:
[^18^F]Gefitinib	Ex vivo*:*	ND	Ex vivo:	ND	ND	Ex vivo:	Ex vivo:
	SUV 0.14 ± 0.05		SUV 0.22 ± 0.09			SUV 0.15 ± 0.10	U87-EGFR
	at 120 min [84]		at 120 min [84]			at 120 min [84]	SUV 0.14 ± 0.06 at 120 min [84]
							Fibrosarcoma:
							3.5%ID/g at 60 min [83]
[^11^C]Erlotinib		Ex vivo:			Ex vivo:		Ex vivo:
		1.62 ± 0.47%ID/g			3.66 ± 0.14%ID/g		NCI358: 0.69 ± 0.11%ID/g at 60 min [95]
		at 60 min [95]			at 60 min [95]		
	In vivo:	In vivo:	In vivo:	In vivo:	In vivo:	In vivo:	In vivo:
	SUV 0.51 ± 0.56	T/NT 1.0 ± 0.3	SUV 0.43 ± 0.01 at 12 min [97]	SUV 0.45 ± 0.31 at 90 min [94]	SUV 0.91 ± 0.60 at 90 min [94]	SUV 0.33 ± 0.03	SW620 (*n* = 1): SUV of 0.28 [94]
	at 90 min [94]	at 60–90 min [96]	SUV 0.33 ± 0.09 at 60 min [97]		SUV 0.60 ± 0.01 at 12 min [97]	at 12 min [97]	U87ΔEGFRvIII: SUV of 0.46 ± 0.29 [94]
					SUV 0.71 ± 0.07 at 60 min [97]	SUV 0.21 ± 0.03	
					3.2 ± 0.3%ID/g at 25 min [96]	at 60 min [97]	QG56*:* SUV 0.34 ± 0.04 at 12 min [97]
					T/NT 1.9 ± 0.5 at 60–90 min [96]	T/NT 1.0 ± 0.3 at 60–90 min [96]	SUV 0.20 ± 0.01 at 60 min [97] IC_50_: 8.9 µM [97]
					V_T_ 0.96 ± 0.15 in 10–60 min interval [88]		A431: V_T_ 0.75 ± 0.06 in 10–60 min [88]
			IC_50_: 40nM [97]		IC_50_: 28.5 ± 4.5nM [88]	IC_50_: 4.3 µM [97]	HCC827_ERLO_: V_T_ 1.05 ± 0.26 in 10–60 min, IC_50_: 9.8 ± 7.4µM
					IC_50_: 4 nM [97]		HCC827_EPR_: V_T_ 1.00 ± 0.15 in 10–60 min, IC_50_: 5.4 ± 2.3µM [88]
[^18^F]Afatinib	ND	Ex vivo:	ND	ND	Ex vivo:		ND
		2.17 %ID/g at 5 min,			1.56 %ID/g at 5 min,		
		T/M 6.37 at 120 min			T/M 3.83 at 120 min		
		[91]			[91]		
		In vivo:			In vivo:	In vivo:	
		T/NT 1.5 ± 0.3			T/NT 2.3 ± 0.4	T/NT 0.8 ± 0.2	
		at 90–120 min [96]			at 90–120 min [96]	at 90–120 min [96]	
					1.2 ± 0.2%ID/g at 10 min [96]		

**Table 7:** IC_50_ values in purified EGFR, EGFR mutated, HER2, and HER4 kinases.

Inhibitor	EGFR Wild-Type	EGFR L858R	EGFR D746–750	HER2	HER4
PD153035	29 ± 5.1 pM			2.3 µM	
Gefitinib	33 nM [114] 3 nM [20,115]	0.8 nM [20]		3.7–10 µM [114] 1.83 µM [20] 343 nM [115]	476 nM [115]
Erlotinib	0.56 nM [115] 0.25 nM [116]	0.36 nM [116]	0.41 nM [116]	512 nM [115] 22 nM [116]	790 nM [115] 265 nM [116]
Afatinib	0.5 nM [20]	0.4 nM [20]		14 nM [20]	
Osimertinib	1.2 nM [116]	2.1 nM [116]	1.2 nM [116]	1.69 nM [116]	3.06 nM [116]

## Data Availability

Not applicable.
